# Process evaluation of an intervention to improve access to injectable contraceptive services through patent medicine vendors in Nigeria: a mixed methods study

**DOI:** 10.1186/s40545-021-00336-5

**Published:** 2021-11-16

**Authors:** Mojisola Morenike Oluwasanu, Ayodeji Matthew Adebayo, Faizah Tosin Okunade, Olayinka Ajayi, Akinwumi Oyewole Akindele, John Stanback, Ademola Johnson Ajuwon

**Affiliations:** 1 Department of Health Promotion and Education, Faculty of Public Health, College of Medicine, University of Ibadan, Ibadan, Nigeria; 2 Department of Community Medicine, Faculty of Clinical Sciences, College of Medicine, University of Ibadan, Ibadan, Nigeria; 3 Sight Savers International, Kaduna, Nigeria; 4 Department of Community Medicine, University College Hospital, Ibadan, Nigeria; 5 FHI 360, Durham, NC USA

**Keywords:** Injectable contraceptives, Patent medicine vendors, Process evaluation, Family planning, Access to healthcare

## Abstract

**Background:**

The low utilisation of modern contraceptives in many low- and middle-income countries remains a challenge. Patent medicine vendors (PMVs) that operate in the informal health sector, have the potential to address this challenge. Between 2015 and 2018, the Population Council, in collaboration with the Federal and State Ministries of Health and the Pharmacy Council of Nigeria, trained PMVs in six states to deliver injectable contraceptive services. Outcome evaluation demonstrated increased client uptake of injectable contraceptive services; however, there is limited information on how and why the intervention influenced outcomes. This study was conducted to elucidate the processes and mechanism through which the previous intervention influenced women’s utilisation of injectable contraceptive services.

**Methods:**

The study utilised a mixed methods, convergent parallel design guided by the UK Medical Research Council framework. Quantitative data were obtained from 140 trained PMVs and 145 of their clients in three states and 27 in-depth interviews were conducted among relevant stakeholders. The quantitative data were analysed descriptively, while the qualitative data were analysed thematically.

**Results:**

The results revealed that even after the completion of the PMV study which had a time-bound government waiver for injectable contraceptive service provision by PMVs, they continued to stock and provide injectables in response to the needs of their clients contrary to the current legislation which prohibits this. The causal mechanism that influenced women’s utilisation of injectable contraceptives were the initial training that the PMV received; the favourable regulatory environment as demonstrated in the approval provided by government for PMVs to provide injectable contraceptives for the duration of the study; and the satisfaction and the confidence the female clients had developed in the ability of the PMVs to serve them. However, there were gaps with regards to the consistent supply of quality injectable contraceptive commodities and in PMVs use of job aids. Referral and linkages to government or private-owned facilities were also sub-optimal.

**Conclusion:**

PMVs continue to play important roles in family planning service provision; this underscores the need to formalize and scale-up this intervention to aid their integral roles coupled with multi-faceted initiatives to enhance the quality of their services.

**Supplementary Information:**

The online version contains supplementary material available at 10.1186/s40545-021-00336-5.

## Background

The low uptake of contraceptives in many low- and middle-income countries (LMICs) remains a serious burden, contributing to maternal mortality [1, 2]. Despite decades of interventions to improve the contraceptive prevalence rate in Nigeria, utilisation remains persistently low with only 10% of currently married women using a modern method [3, 4]. Factors accounting for this include individual, cultural, legal, policy and institutional factors which limit women’s access and utilisation of injectables and other contraceptives [5–10].

In Nigeria, injectables, namely, Depo-Provera, Noristerat and Sayana Press, are popular contraceptive methods, accounting for about 30% of all types of contraception used by women [2]. Currently, the private sector provides 23.1% of the injectable contraceptive services in the country with the patent medicine stores accounting for 9% of this [4]. The patent medicine vendors (PMVs) are a group of frontline health workers who operate in the informal sector of the Nigerian health system [11]. They are the primary source of contraceptives because of the convenient locations of their shops, flexible operation hours, affordability of their services, availability of reliable drug stocks and confidentiality [12]. Although the pharmacy laws prohibit PMVs from selling and administering injectable contraceptives [13, 14] data from the latest (2019) Demographic Health Survey (DHS) [4] and other research [12] confirm that PMVs provide these services. Similar findings have been observed in other sub-Saharan Africa countries [15–18]. Thus, there is a growing recognition of the need to integrate this category of health workers into the formal health care system if the country is to meet its target of universal health coverage and increasing the contraceptive prevalence rate [19].

In 2015, the Population Council, an international non-governmental organisation, in collaboration with the Federal and State Ministries of Health, began a two-phase, pilot implementation research project to train 381 PMVs to deliver injectable contraceptive services in drug shops in six states; one each in the six geopolitical region of Nigeria [15].

Outcome evaluation of this intervention demonstrated enhanced knowledge and skills of PMVs on injectable contraceptive services provision as well as increased uptake and acceptability by female clients [15, 20]. However, when evaluating  complex interventions, it is necessary to understand the specific processes and causal mechanisms through which implementation strategies influence outcomes [21, 22]. Gaining insights into the mechanisms that lead to success or failure can improve the selection of the most appropriate implementation strategies, increase cost effectiveness, and guide scale-up of evidence-based interventions. In view of this, the aim of this process evaluation study was to elucidate the processes and mechanisms of change of a previous implementation research designed to assess the feasibility of PMVs administering all forms of progestin-only injectable contraceptives, namely, Depo-Provera and Sayana Press in six states in Nigeria.

## Methods

### Study design

A mixed methods convergent parallel design guided by the UK Medical Research Council (MRC) framework was used to assess the implementation processes and mechanisms of change. The UK Medical Research Council framework posits that the outcome of a complex intervention is influenced by the interactions between the implementation processes, context, and mechanisms. We applied a theory of change articulating the project**’**s hypothesised pathways to intervention outcomes and utilised both quantitative and qualitative primary and secondary data from a wide range of sources [15, 20, 23] to assess implementation, and understand causal mechanisms. This approach was aimed at triangulation of data which enriched the interpretation of the results. Data from both sources were analysed separately and results integrated to generate conclusions.

### Study sites

The study was conducted in 2020 in 3 states from three geo-political zones (Oyo state, South-West; Cross-Rivers state, South-South; Kaduna state, North-Central) out of the six geo-political zones in where the previous intervention was implemented. Data were collected from eleven (11) LGAs from the three (3) states; from Cross-Rivers-Calabar Municipal, Calabar South, Akpabuyo and Odukpani; from Oyo-Afijio, Ona-Ara and Egbeda; from Kaduna-Igbadi, Kaduna South, Kaduna North and Chikun.

### Conceptual framework

The UK Medical Research Council framework [24, 25] guided the selection of key constructs and this was used in developing the evaluation questions in this study. Constructs of this framework which were assessed are Context, Description of intervention and its causal assumptions, Implementation process, Mechanism of impact and Outcomes.

The evaluation of the implementation process was guided by a critical analysis of two domains, namely, fidelity (extent to which the intervention activities were delivered as intended) and dose (what components of the intervention were delivered most or less to the PMVs). These helped to determine if the project outcomes can be attributed to the intervention strategies or if there was implementation failure.

**Fig. 1:**
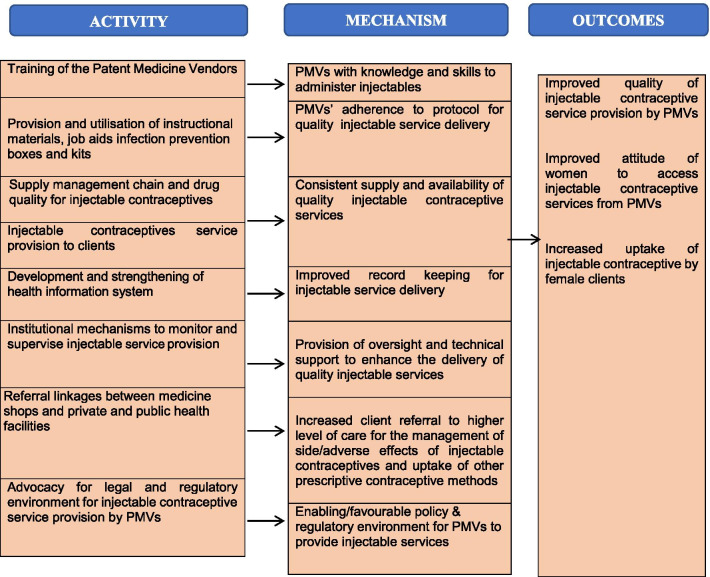
Theory of change showing mechanisms linking inputs and outcomes

The “mechanisms of change/impact” which focuses on participants’ responses to programme services was also assessed [26]. Mechanism evaluation helps to determine the intervention’s pathways of change from the project inputs to the project outcomes. The mechanism evaluation was guided by the Theory of Change developed by 20 professionals drawn from a broad range of family planning experts including government agencies, family planning service providers, non-governmental organisations and PMVs during a stakeholders’ meeting in Ibadan. Nigeria (Fig. 1) [26–28]. The ToC describes how interventions can bring about long-term outcomes through a logical sequence of intermediate outcomes (also known as mediators) and has been used to design and measure the impact of public health programmes in several countries [28].

Specifically, we assessed whether the intended mechanisms worked as designed and how the intervention concepts and ideas were adopted or adapted. The information obtained during this activity guided the adaptation/development of qualitative and quantitative tools used for data collection. We highlighted seven key mechanisms through which the programme activities were hypothesised and linked with outcome, as summarised in Fig. 1 and this guided the presentation of the results. Figure 1 outlines the specific activity which links with a mechanism and the outcome. Key interactions which are particularly important are the training of the patent medicine vendors and the legal and regulatory environment for injectable contraceptive service provision by PMVs.

### Tools for data collection

Quantitative and qualitative instruments (Additional files 1, 2, 3) were used for data collection. The tools comprised of closed and open-ended questions which explored the implementation processes (training, supervisory visits, contraceptive supply, infection prevention, Information, Education and Communication materials and logistical management), causal mechanisms and how the training interventions influenced women’s uptake of injectable contraceptives. For the PMVs and their clients, the English version of the questionnaires and in-depth interview guide (PMVs only) were translated into Yoruba and Hausa Languages and administered in the preferred language of the respondents.

### Training of research assistants and pre-testing of tools

Prior to the commencement of data collection activities, the research team conducted a 2-day training for 6 research assistants per states (total 18) who had postgraduate training in the social sciences or public health and majority were females. The questionnaire and interview guides were pre-tested in Ibadan North Local Government Area in Oyo state prior to the conduct of the actual study.

### Quantitative data collection

A multistage sampling technique was used to select 140 trained PMVs from three of the six project states (50, 44 and 46 in Oyo, Kaduna, and Cross-Rivers states, respectively) taking into cognizance the urban–rural distribution. PMVs were interviewed in their shops using a questionnaire (Additional file 1) and each interview lasted an average of 25 minutes.

For female clients, the research assistants used the de-identified injectable service records of each trained PMV to identify eligible study participants. Permission was obtained from each of the selected PMVs to use the de-identified injectable service records. Eligible women were identified using the following four criteria:

- *utilisation of injectable contraceptive services from the PMVs occurred in 6 months preceding survey*
- *Aged at least 18 years*
- *Had a mobile phone number*
- *Willing to participate in the study*

The research team adopted several measures to protect the privacy and confidentiality of the female clients during recruitment. Specifically, the PMVs were requested to call the clients and explain the purpose of the current study and its linkage with the previous pilot implementation research  project. If a client agreed to participate, the PMVs sent the informed consent form to the woman. On receiving the signed consent forms, the trained research assistants called eligible clients to confirm their willingness and schedule the telephone interview. The telephone interview sessions were conducted using a questionnaire (Additional file 2) and lasted an average of 15 minutes.

### Qualitative data collection

Twenty-seven in-depth interviews were conducted among PMVs, officials of the association of PMVs, health workers, government regulatory officials and a programme implementer using pre-tested interview guides (Additional file 3). The interviews were conducted until data saturation was achieved. Respondents were purposively selected; for the interview with health workers, two public or private health facilities that provided family planning services and were proximal (2–5 kilometres) to the store of a selected PMV were randomly selected and interviewed. Government officials from the family planning and pharmacy departments of the Ministries of Health, staff of the Pharmacy Council of Nigeria and the representative of the implementing organisation who were involved in the implementation of the previous study were also interviewed (see Table 1 for details).

**Table 1:** Variable definition for the quantitative data

Variable	Definition	Categorisation
Knowledge of progestin-only injectable contraceptives (Depo-Provera, Noristerat and Sayana Press)	A computation of three knowledge questions for progestin-only injectable contraceptives (Depo-Provera, Noristerat and Sayana Press): (1) the type of device used to administer the progestin-only injectable contraceptive; (2) the site where the progestin-only injectable contraceptive can be administered on the body; and (3) the reinjection frequency of the progestin-only injectable contraceptive [15]	A dichotomised variable was created and coded as 1 if the PMV answered all aspects of each question correctly, and 0 if the PMV answered any part of the three questions incorrectly
Specific injectable contraceptive knowledge variable	A computation of knowledge questions on the specific contraceptive specifically: (1) the type of device used to administer the specific injectable contraceptive; (2) the site where specific injectable contraceptive can be administered on the body; and (iii) the reinjection frequency of specific injectable contraceptive [15]	A dichotomised variable was created and coded as 1 if the PPMV answered all aspects of each question correctly, and 0 if the PPMV answered any part of the three questions incorrectly
Side effect	This was PMVs’ spontaneous response to the question “what are the common side effects of progestin-only injectable contraceptives.” The seven possible responses include: weight gain, change in menstruation, delayed return to fertility, dizziness, decrease in sex drive, mild skin irritation, and headaches	A dichotomised variable was created and coded as 1 (i.e., good) if the PMV named at least 4 of the 7 common side effects and 0 (i.e., poor) if the PMV named less than 4 of these side effects

The key informant interviews were conducted by the trained research assistants or some members of the research team—MMO, AMA, AOA, FTO and OA, who all had postgraduate training/degrees in public health and an average of 15-year experience in qualitative research studies. The members of the research team who conducted the interviews (three females and two males) were academic/research staff at tertiary academic/health institutions or non-governmental organisations in Nigeria with a deep understanding of the cultural and public health context of the study settings. The average duration of the KII and IDI was 45 minutes. Face-to-face interviews were conducted at the health facilities, offices and PMV shops in venues which were free of distraction and noise and offered privacy; however, the interview with the program implementer was conducted on the telephone. Informed consent was obtained from all participants, and digital recorders were used to document the interviews. The interviewees were provided with detailed information on the objectives of the study and assured of confidentiality.

### Data analysis

The quantitative data were analysed descriptively to generate indicators of implementation and provide insights on the context surrounding the intervention and mechanisms of change. The variable definition for the quantitative data is outlined in Table 1 and these were analysed using SPSS version 21.

For the qualitative data, steps for data analysis included transcription of the digital recordings, reading the transcripts to get acquainted with the data, review meetings by the researchers to discuss the interview transcripts prior to coding to identify points of agreement and resolve disagreements and coding using a pre-determined coding frame. The coding frame had codes such as “training”, “commodity supplies” “referral”, “record keeping” etc. Thereafter, we sorted the codes into themes/clusters in line with the constructs of the UK MRC framework. The qualitative interview transcripts were coded using NVIVO 12.0 software. Data analysis and interpretation were done by three coders to ensure the accuracy of the findings in terms of theme identification, understanding of concepts used and clarification of the idiomatic and metaphoric language. Subsequently, there was a triangulation of the qualitative and quantitative data and the research team provided oversight supports to ensure the trustworthiness of the data. The Consolidated Criteria for Reporting Qualitative Research (COREQ) checklist was used to report the qualitative research.

### Data integration and synthesis method

The qualitative and quantitative data were integrated and merged [29]. This enhanced understanding of the implementation processes, mechanisms of change and context of the intervention. Themes from the data sets were compared to identify differences or commonalities and subsequently, there was a narrative description and presentation of both the qualitative and quantitative findings by themes [29].

### Ethical considerations

The Joint University of Ibadan/University College Hospital Ethics Review Committee and the Ethics Review Committee of the World Health Organisation provided approval for the conduct of the study. Informed consent was obtained from each study participant using a structured written consent form.

## Results

### Demographic characteristic of the PMVs and their clients

For the quantitative data, there were more male PMVs (52.1%) than females (47.9%). The highest proportion (56.4%) were aged 40–59 years (Table 2). The mean age of clients of PMVs was 33.3 ± 6.3 years, the majority (> 70%) were aged 25–39 years and 86.7% were married. Completion of secondary school certificate examination was the highest reported level of education (45.5%) (Table 2).

**Table 2:** Socio-demographic characteristic of the PMVs and female clients

Variables	Oyo State (*N* = 50) *n* (%)	Kaduna State (*N* = 44) *n* (%)	Cross River State (*N* = 46) *n* (%)	Total (*N* = 140) *n* (%)
Socio-demographic characteristics of PMVs				
Sex				
Male	19 (38.0)	24 (54.5)	30 (65.2)	73 (52.1)
Female	31 (62.0)	20 (45.5)	16 (34.8)	67 (47.9)
Age group (years)				
≤ 24	0 (0.0)	2 (4.5)	3 (6.5)	5 (3.6)
25–39	7 (14.0)	21 (47.7)	22 (47.8)	50 (35.7)
40–59	41 (82.0)	19 (43.2)	19 (41.3)	79 (56.4)
≥ 60	2 (4.0)	2 (4.5)	2 (4.3)	6 (4.3)
Mean ± SD	46.40 ± 8.09	39.11 ± 8.49	40.35 ± 11.69	42.12 ± 10.00
Socio-demographic characteristics of female clients				
Age group (years)				
≤ 24	2 (4.4)	5 (11.1)	6 (11.1)	13 (9.0)
25–39	34 (75.6)	32 (71.1)	39 (72.2)	105 (72.9)
≥ 40	10 (20.0)	8 (17.8)	9 (16.7)	27 (18.1)
Mean age	34.0 ± 5.7	33.1 ± 6.1	32.6 ± 6.9	33.3 ± 6.3
Marital status				
Married	45 (97.8)	45 (100.0)	36 (66.0)	126 (86.7)
Never married	1 (2.2)	0 (0.0)	12 (22.7)	13 (9.1)
Not married, in-union	0 (0.0)	0 (0.0)	3 (5.7)	3 (2.1)
Separated/divorced	0 (0.0)	0 (0.0)	3 (5.6)	3 (2.1)
Highest level of education				
Koranic/no formal education	0 (0.0)	2 (4.5)	2 (3.8)	4 (2.8)
Primary school leaving certificate	11 (23.9)	10 (20.5)	7 (13.2)	28 (19.3)
West Africa School Certificate/Senior School Certificate Examination	26 (56.6)	14 (31.8)	26 (49.1)	66 (45.5)
Ordinary National Diploma/General Certificate Examination	6 (13.0)	13 (29.6)	7 (13.2)	26 (17.9)
Higher National Diploma/Bachelor degree	3 (6.5)	6 (13.6)	12 (20.7)	21(14.5)

For the qualitative data, 11 females and 16 males were interviewed. There were eight interviewees from Oyo State, 10 from Cross rivers and nine in Kaduna state (Table 3).

**Table 3:** Distribution of respondents selected for the qualitative interviews by sites

State	PMV	Health Worker	NAPPMED official	PCN official	Government official (Family Planning (FP) coordinator	Programme Implementer	Total
Oyo State	3	2	1	–	1	1	8
Cross Rivers	4	3	1	1	1	–	10
Kaduna State	3	2	2	1	1	–	9
Total	10	7	4	2	3	1	27

### Process evaluation and causal mechanisms which influenced injectable contraceptive service provision

#### Training of the patent medicine vendors

Findings indicate that the training had a high level of fidelity and was implemented as expected. Trainers used WHO manuals on injectable contraceptives and an adapted curriculum developed by PATH and FHI 360 which was used to train Community Health Extension Workers on injectable contraceptive services in Uganda. This was approved by the Federal Ministry of Health.

The proposed mechanism was that the training would improve PMVs’ knowledge, skills, and motivation to provide injectable contraceptive services. According to views of the participants, the training programmes were competency-based, involving both didactic and practical sessions with stringent thresholds set for graduation and certification.

*It was a quality training; an intensive training and you just must learn before you can be given the certificate to practice (PMV_ Kaduna)*

*…. the trainers were highly educated and disciplined, the way they guided us is the way we follow up with our clients (PMV_ Cross river)*

**Fig. 2:**
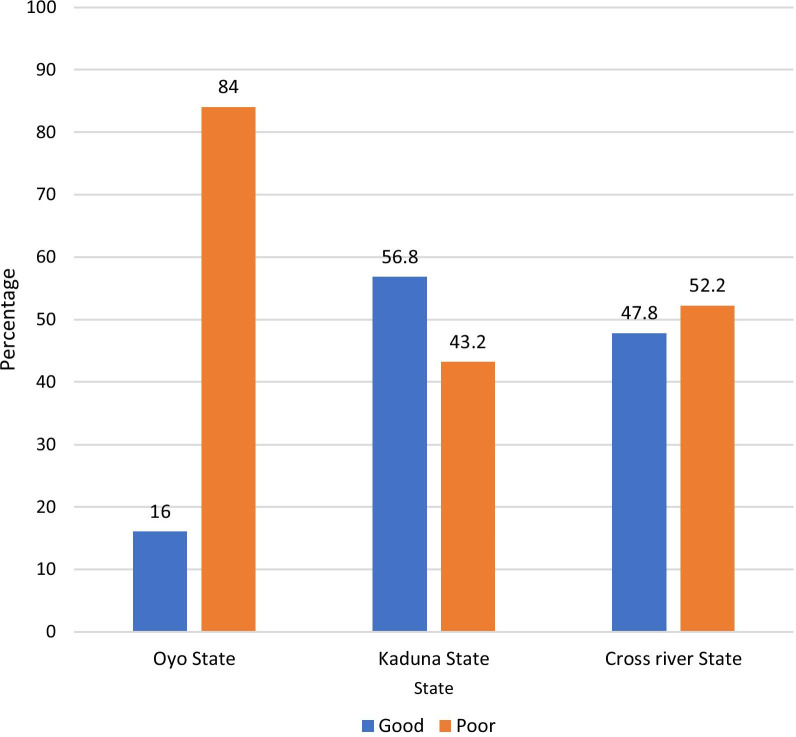
PMVs’ knowledge of side effects of injectables

Similar to the outcome evaluation of the pilot implementation research, findings from the current study showed that PMVs in each of the study sites were knowledgeable about each of the injectable contraceptives (Table 4). The complete knowledge of Depo-Provera was highest (96.4%); however, the knowledge of side effects was generally poor (60.7%) across all study sites (Fig. 2).

**Table 4:** PMVs’ knowledge on injectable contraceptives and perception of the training

Variables	Oyo State (*N* = 50) *n* (%)	Kaduna State (*N* = 44) *n* (%)	Cross river State (*N* = 46) *n* (%)	Total (*N* = 140) *n* (%)
PMVs’ knowledge on injectable contraceptives:				
* a. Depo-Provera*				
Complete	48 (96.0)	42 (95.5)	45 (97.8)	135 (96.4)
Incomplete	2 (4.0)	2 (4.5)	1 (2.2)	5 (3.6)
* b. Noristerat*				
Complete	36 (72.0)	41 (93.2)	44 (95.7)	121 (86.4)
Incomplete	14 (28.0)	3 (6.8)	2 (4.3)	(13.6)
* c. Sayana Press*				
Complete	40 (80.0)	37 (84.1)	40 (87.0)	117 (83.6)
Incomplete	10 (20.0)	7 (15.9)	6 (13.0)	23 (16.4)
Training improved knowledge and skills in injectable service provision				
Strongly agree	43 (86.0)	37 (81.8)	41 (89.1)	120 (85.7)
Agree	7 (14.0)	7 (18.2)	5 (10.9)	20 (14.3)
Training improved knowledge and skills in FP counselling				
Strongly agree	44 (88.0)	35 (79.5)	40 (87.0)	119 (85.0)
Agree	6 (12.0)	9 (20.5)	6 (13.0)	21 (15.0)
Perception of how training was				
Very easy/Easy	44 (88.0)	39 (88.6)	37 (80.5)	120(85.8)
Moderate	5 (10.0)	5 (11.4)	7 (15.2)	17 (12.1)
Difficult	1 (2.0)	0 (0.0)	2 (4.3)	3 (2.1)
Participation in any other training on FP				
No	41 (82.0)	13 (29.5)	31 (67.4)	85 (60.7)
Yes	9 (18.0)	31 (70.5)	15 (32.6)	55 (39.3)
Source of training (*N* = 56)				
Governmental	3 (30.0)	0 (0.0)	1 (6.7)	4 (7.1)
Non-governmental	7 (70.0)	31 (100.0)	14 (93.3)	52 (92.9)
Types of NGOs that trained PMVs (*N* = 52)				
NURHI	2 (28.6)	21 (67.7)	0 (0.0)	23 (44.2)
SFH	4 (57.1)	7 (22.6)	3 (21.4)	14 (26.9)
PATHFINDER	0 (0.0)	0 (0.0)	8 (57.1)	8 (15.4)
Others*	1 (14.3)	3 (9.7)	3 (21.4)	7 (13.5)

*Others UNICEF, USAID, Irene and Mobil, ARFH, FHI 360, and Population Council of Nigeria

The majority (85.7%) of the PMVs reported that the training by the Population Council improved their knowledge, skills in injectable contraceptive counselling and service provision including how to address concerns about side effects and contraceptive failures as shown below:

*After the training, there is no more doubts and fear……, before now, we were usually afraid about the result (contraceptive failure, side effects) (PMV_Oyo)*

#### Provision and utilisation of instructional materials and job aids

A key adaption in the second phase was the provision of instructional materials and job aids to trained PMVs. This was based on lessons learnt during the follow-up monitoring and evaluation which revealed that PMVs did not provide detailed information to clients on the side effects of injectables during their counselling sessions. Subsequently, all PMVs trained in the first and second phases were provided instructional materials and job aids (*Balanced Counselling Strategy (BCS) plus cards, Medical Eligibility Criteria (MEC) Wheel, Family Health International (FHI) Checklist and FHI Reinjection job aide).* The quote below underscores this finding:

*Well we discovered that PMVs were not really telling the women all the ranges of side effects that the women could experience when they are using injectables. The question we asked the women during the [follow-up interviews] showed that PMVs all remembered common side effects such as headache and irregular bleeding, but other side effects were not mentioned ………because PMVs are not very educated they may not remember all those kind of things so we choose to develop job aids that PMVs can use (Programme Implementer).*

Findings from the current study showed that only 27.9% PMVs reported the use of these aids. The findings on the sub-optimal use of job aids was supported by qualitative data as expressed below:

*Yes, some of them were keeping the instructional materials in their drawers, some were not using it but during the monitoring [visits] they were retrained and encouraged to continue usage (Government regulatory agency Kaduna)*

A strategy devised to address this gap was the constant reminder on the importance of the job aids for the delivery of effective and safe injectable contraceptive services during the monitoring and follow-up visits.

#### Supply management chain and drug quality for injectable contraceptives

The supply of injectable contraceptives was a component of the intervention which had some gaps. Efforts were made to establish a reliable commodity supply mechanism by providing a seed stock of injectables to the PMVs and establishing re-supply linkages with credible organisations. These organisations were the Deep K. Tyagi Foundation and the Society for Family Health both based in Nigeria. Deep K. Tyagi is an international, non-profit, private organisation which utilizes social marketing to address the family planning needs of several countries including Nigerians, while the Society for Family Health is a Nigerian non-government organisation which works in partnership with communities, government and the private sector to address several public health challenges. The expected mechanism was that, linking the PMVs to suppliers would prevent injectable contraceptive stockout and ensure the availability of affordable contraceptives.

**Fig. 3:**
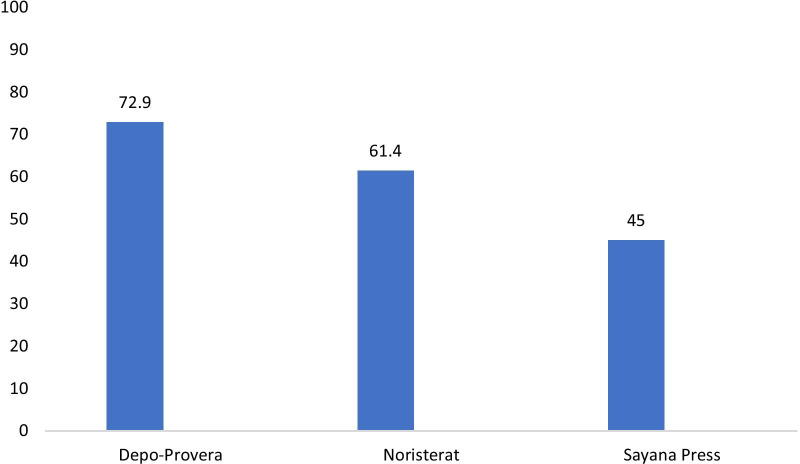
Availability of injectable contraceptives in the PMVs’ stores

Figure 3 shows the availability and pattern of injectable contraceptives among PMVs. Overall, Depo-Provera was in stock among 72.9% of the PMVs, followed by Noristerat (61.4%) and Sayana Press (45.0%). This varied by state (see Table 5). As at the time of the conduct of this study, the cost for the provision of the injectables to clients was as follows: Depo-Provera (N336 ± N223; USD$0.93 ± USD$0.62), Noristerat (N306 ± N206; USD$0.85 ± USD$0.57) and Sayana Press (438 ± 265; USD$1.22 ± USD$0.74). Of interest is Sayana Press—an injectable contraceptive newly introduced to the Nigerian market at the time of the intervention. This product was marketed by DKT whose staff made a presentation on its administration, side and adverse effects and promised to supply the stores as stated in this quote:

*……for the study we wanted to be sure that the PMVs were using correct contraceptive methods so that we do not have excess failure rate. You know, if we don’t provide them with reliable supply, they can be getting it from outside market… So, what we did was that, we worked with Society for Family Health and DKT. ………we gave all PMVs a seed stock and we linked them up with SFH for additional supply; so that was what we did for the Noristerat and Depo-Provera. For Sayana Press we invited DKT. DKT attended all the training sessions and DKT was given approximately 1hour to present the Sayana Press and to teach the PMVs directly how to administer Sayana Press ….. and then they gave them their contact number so that whenever they need commodity supplies, they can call (Programme Implementer).*

**Table 5:** Availability and pattern of injectable contraceptives among PMVs

Variables	Oyo State (*N* = 50) *n* (%)	Kaduna State (*N* = 44) *n* (%)	Cross river State (*N* = 46) *n* (%)	Total (*N* = 140) *n* (%)
Currently have Depo-Provera in stock				
No	10 (20.0)	13 (29.5)	15 (32.6)	38 (27.1)
Yes	40 (80.0)	31 (70.5)	31 (67.4)	102 (72.9)
Currently have Noristerat in stock				
No	24 (48.0)	14 (31.8)	16 (34.8)	54 (38.6)
Yes	26 (52.0)	30 (68.2)	30 (65.2)	86 (61.4)
Currently have Sayana Press in stock				
No	20 (40.0)	30 (68.2)	27 (58.7)	77 (55.0)
Yes	30 (60.0)	14 (31.8)	19 (41.3)	63 (45.0)
Number of Depo-Provera given last month				
None	19 (38.0)	11 (25.0)	26 (56.5)	56 (40.0)
≥ 1 unit	31 (62.0)	33 (75.0)	20 (43.5)	84 (60.0)
Number of Noristerat given last month				
None	35 (70.0)	12 (27.3)	30 (65.2)	77 (55.0)
≥ 1 unit	15 (30.0)	32 (72.7)	16 (34.8)	63 (45.0)
Number of Sayana Press given last month				
None	29 (58.0)	30 (68.2)	41 (89.1)	100 (71.4)
≥ 1 unit	21 (42.0)	14 (31.8)	5 (10.9)	40 (28.6)

However, there was a challenge sustaining supply of Sayana Press; DKT supplied the commodities but the supply was infrequent.

*We were told that some people will be supplying us, and we didn’t see them. It was only DKT that supplied us with commodities, and they were not frequent. (PMV_Oyo)*

This had implications for injectable service delivery. Users who were already introduced to the Sayana Press could no longer access the services, because it was unavailable or expensive as expressed below:

*Our only challenge was the unavailability and high cost of Sayana Press. You know a lot of people after we have introduced Sayana Press to them, they already liked it and were used to it and all of a sudden it became scarce and expensive……some clients are always scared of needles but Sayana Press needle is really small (PMV_Oyo)*

To address this challenge, most of the PMVs obtained their commodities from the open market or pharmacies.

#### Injectable contraceptives service provision to clients

Overall, 88.4% of the clients were current users of injectable contraceptives. Of this proportion, 93.8% received their most recent injections. Among the very few who were not recent users, the highest proportion of the clients reported stoppage due to the side effects (31.8%), followed by not having enough time to go back (27.3%). Among the current injectable contraceptive users, virtually all (99.2%) received their most recent injections from the same PMV as previous injections (Table 6).

**Table 6:** Current use of injectable contraceptives among clients of PMVs

Variables	Oyo (*N* = 46) *n* (%)	Kaduna (*N* = 45) *n* (%)	Cross River (*N* = 54) *n* (%)	Total (*N* = 145) *n* (%)
Current use of injectable contraceptives				
Yes	45 (98.8)	43 (95.6)	41 (75.9)	129 (88.4)
No	1 (2.2)	2 (4.4)	13 (24.1)	17 (11.6)
Received most recent injections				
Yes	42 (93.3)	42 (97.7)	37 (90.2)	121 (93.8)
No	3 (6.7)	1 (2.3)	4 (9.8)	8 (6.2)
Reasons for not currently using injections (*N* = 25)				
Forgot to go back	1 (20.0)	0 (0.0)	0 (0.0)	1 (4.0)
Did not have time to go back	1 (20.0)	1 (33.3)	4 (25.0)	6 (24.0)
Did not like the method	1 (20.0)	1 (33.3)	0 (0.0)	2 (8.0)
Stopped because of side effects	1 (20.0)	1 (33.4)	5 (31.2)	7 (28.0)
Stopped because I wanted to become pregnant	1 (20.0)	0 (0.0)	0 (0.0)	1 (4.0)
Stopped because I am pregnant	1 (20.0)	0 (0.0)	2 (12.5)	3 (12.0)
Stopped because of partner	0 (0.0)	0 (0.0)	2 (12.5)	2 (8.0)
Others*	0 (0.0)	0 (0.0)	3 (18.8)	3 (12.0)
Type of injectable contraceptives currently used				
Depo-Provera	29 (69.0)	20 (47.6)	23 (62.2)	72 (59.5)
Noristerat	2 (4.8)	14 (33.3)	10 (27.0)	26 (21.5)
Sayana Press	11 (26.2)	7 (16.7)	3 (8.1)	21 (17.4)
Don’t know	0 (0.0)	1 (2.4)	1 (2.7)	2 (1.6)
Where injectable contraceptive was received recently				
Same PMV as last injection	41 (97.6)	42 (100.0)	37 (100.0)	120 (99.2)
Public health facility	1 (2.4)	0 (0.0)	0 (0.0)	1 (0.8)
Type of family planning services received from PMVs last visit				
Purchased injectable and referred facility for injection	0 (0.0)	0 (0.0)	1 (2.7)	1 (0.8)
Purchased and received injectable shot	34 (81.0)	42 (100.0)	36 (97.3)	112 (92.6)
Referred to facility for injectable	8 (19.0)	0 (0.0)	0 (0.0)	8 (8.6)

*Lack of money (1), Stopped for no reason (1), Not convenient going every 3 months (1)

Qualitative data also revealed that there was an increase in the utilisation of injectable contraceptive services from the PMV stores as expressed below:

*……the difference is very, very clear, ah! before in a month, we only have two or three women that come for family planning in the shop, but after the training, a lot of them come, they are receiving information from their neighbours, from their friends that they should go to the PMV shops to do family planning, it [ getting injectables] does not give any problem (PMV_Kaduna)*

These qualitative views resonated with the quantitative data collected from the female injectable users.

### Clients’ perception and satisfaction with PMV services

The clients perceived the PMVs to be very knowledgeable (95%) and they got all the services they needed from the PMVs (Table 7). Similarly, they all reported they were satisfied with the services received from the PMVs. Reasons given for satisfaction with the services received include perception that the PMVs is knowledgeable (28.1%), display of professionalism (25.6%) and friendly and caring nature of the PMVs (17.4%). Majority (97.9%) reported they were comfortable recommending PMV to someone who needs injectable contraception (Table 7).

**Table 7:** Clients’ perception and satisfaction about PMV services

Variables	Oyo (*N* = 46) *n* (%)	Kaduna (*N* = 45) *n* (%)	Cross River (*N* = 54) *n* (%)	Total (*N* = 145) *n* (%)
Felt PMV was knowledgeable about injectable contraceptives				
Very knowledgeable	37 (88.1)	42 (100.0)	36 (97.3)	115 (95.0)
Somewhat knowledgeable	4 (9.5)	0 (0.0)	1 (2.7)	5 (4.2)
Neutral	1 (2.4)	0 (0.0)	0 (0.0)	1 (0.8)
Got all the services I needed from the PMV	42 (100.0)	42 (100.0)	37 (100.0)	121 (100.0)
Level of satisfaction with the services received				
Satisfied	42 (100.0)	42 (100.0)	37 (100.0)	121 (100.0)
Reasons for satisfaction				
Knowledgeable/Answer all questions/Gave good advice	11 (26.2)	16 (38.1)	7 (18.9)	34 (28.1)
Professionalism/good services/quality of services	14 (33.3)	7 (16.7)	10 (27.0)	31 (25.6)
Friendly/Sense of humor/comfortable to interact with/caring	8 (19.0)	9 (21.4)	4 (10.8)	21 (17.4)
No side effects/no challenges/No problem since using PMV	8 (19.0)	1 (2.4)	7 (18.9)	16 (13.2)
Confidentiality/Privacy/Trustworthy	1 (2.4)	5 (11.9)	0 (0.0)	6 (5.0)
Low cost	0 (0.0)	1 (2.4)	4 (10.8)	5 (4.1)
Proximity	0 (0.0)	2 (4.8)	1 (2.7)	3 (2.5)
Others**	0 (0.0)	1 (2.4)	4 (10.8)	5 (4.1)
Perception about recent services received from the PMVs				
Knowledgeable	27 (71.1)	32 (76.2)	29 (80.6)	88 (75.9)
Cost	1 (2.9)	11 (27.5)	23 (63.9)	35 (31.8)
Hours of operation appears convenient	5 (14.3)	13 (32.5)	14 (38.9)	32 (28.8)
Friendly/relative/neighbour	8 (23.5)	10 (25.0)	14 (38.9)	32 (29.1)
Location is convenient	11 (31.4)	20 (50.0)	15 (42.9)	46 (41.8)
Always has drugs in stock	1(2.9)	2 (5.0)	10 (27.8)	13 (11.8)
I receive all the information I need	5 (14.7)	16 (40.0)	8 (22.2)	29 (26.4)
Will you recommend PMV to someone else who needs the injection				
Yes	46 (100.0)	45 (100.0)	51 (94.4)	142 (97.9)
No	0 (0.0)	0 (0.0)	3 (5.6)	3 (2.1)

**Drug availability, sending reminders, sells other medications, love for injectables, need for FP

#### Institutional mechanisms to monitor and supervise injectable service provision

A key objective of the previous study was to demonstrate evidence on the feasibility of PMVs providing injectable contraceptives and obtain stakeholders’ buy-in for the pilot initiative. To achieve this, an expanded team comprising representatives of relevant stakeholders from the Federal and State Ministries of Health, NAPPMED, Population Council was constituted to monitor the PMVs. In line with the schedule stated in the protocol, supervisory and follow-up visits were conducted in the 1st, 3rd, 5th, and 9th month post training. Quotes below support this multi-stakeholder mechanism for monitoring and supervision:

*So anytime we go for monitoring, all stakeholders were involved…... The federal ministry of health was always there, NAPPMED was always there, the state government family planning coordinator of every state was also there, Population council was also there, the trainers who trained the PMVs were always there so as to ensure that it is a product of team work* (*Programme* *Implementer*).

According to the quantitative data, almost all the PMV trainees strongly agreed that they benefited from the monitoring activities. Qualitative data supports this finding; according to the respondents, this activity was an opportunity for continuous training and the provision of technical support as illustrated in the quote below:

*…. whenever they come, they remind us about what we were taught and how we were supposed to do it; So, it was a kind of reminder (PMV_Kaduna)*

Another interesting finding is the effect of the monitoring visits on the community acceptance of the PMVs and the service they provide as expressed in the quote below.

*…..when they (the expanded monitoring and supervision team] came here and people [community members] saw them, they knew that I am different from all other PMVs .They saw that many special people [implementers and government partners] visit me so they are like wow! this girl! it’s like she knows something and she is so connected (laughs). So, they come to me, they rush to me, in fact in as much as it is family planning in this environment, I am the only one they come to (PMV_Kaduna)*

#### Referral linkages with private and public facilities

PMVs were provided referral forms to refer clients who had side effects or requested other prescriptive contraceptive methods to proximal private or government facilities. From the quantitative data, 62.9% of the PMVs reported ever interacting with the health facilities in their LGAs. According to qualitative findings, referral varied between the states; some health facility had clients referred, while some did not, as shown in the quotes below:

*It was a chemist person (PMV) that called me. They called me to ask what they should do since a client was complaining of certain things….* (Health worker_ Cross River)

*personally, I have not seen their referrals because there is nothing to show referral from PMVs to us, there is no letter, there is no card to show they are coming from PMVs to us in the health centre, there is nothing to identify* *(Health Worker_Oyo).*

A factor responsible for this variation could the limited need for referral as expressed in the quote below:

*We do not have any complication, so we didn’t refer any patient  [client] to the hospital* *(PMV_Kaduna)*

#### Legal and regulatory environment for injectable contraceptive service provision by PMVs

In general, the study facilitated a favorable policy and regulatory environment for the provision of injectable contraceptives by PMVs throughout the duration of the study. Prior to the study, PMVs provided injectable contraceptives but this action was illegal. To address this challenge, the project activities were designed to advocate to the government health agency and other relevant stakeholders to support the pilot testing of this study by granting a waiver to permit trained PMVs to provide injectables for the entire project duration. In addition, the buy-in of relevant stakeholders such as the Federal and States Ministries of Health, Department of Pharmaceutical services and the National Reproductive Technical Working group was obtained after series of interactions and presentations on the goal and objectives of the study. Eventually, the Federal Ministry of Health gave an approval with a proviso that the PMVs should only provide the injectable contraceptives during the duration of the study.

*the Federal Ministry of Health was involved, and State Ministries of Health gave approval. We initially obtained ethical approval from the University of Ibadan …. We also got ethical approval and letter of support from the Federal Ministry of Health and Department of Pharmaceutical Services. So, the approval from the Federal Ministry of Health and the letter of support was accepted by the State Ministry authorities………. and then coordinators of PMVs, NAPPMED and Pharmaceutical Services. The holistic approach was what gave us the support and the leverage to work (Programme Implementer)*

Subsequently, the PMVs were trained and given certificates which served as evidence of their competency and approval from government to administer injectable contraceptives. The respondents attested that the certification helped legitimise their activities and facilitated a favorable environment for service provision as expressed in the quote below:

*we had certificates and we had the opportunity to put it on the wall. When they come [Police and other regulatory bodies] and start saying this and that [querying them for administering injectable contraceptives]; we tell them we were trained on this and we were asked to use it. So, I think the certificate has really helped because without the certificate, I don’t know how we could convince them that we had the right to do this. So, I think it helped (PMV_Kaduna)*

However, the PMVs opined that despite the approval by the Federal and States Ministries of Health, the Pharmacy Council of Nigeria and in some instances, the Department of Pharmaceutical Services at the State Ministry of Health were antagonistic and unsupportive of the initiative. This view was supported by quotes from a PMV and representatives of government regulatory agency as expressed below:

*They [Pharmacy Council of Nigeria] never received us [accepted us]. They antagonised us as being unqualified. [PMV_Oyo]*

*it [the intervention] didn’t go down well with him [Head of Pharmaceutical, Department of Pharmaceutical Services], He said we are trying to dilute the system (Government regulatory agency Cross rivers)*

A key step to engaging with the Nigerian legal framework to ensure the continued provision of injectable services by PMVs was the presentation of the findings of the feasibility study to the National Council on Health-*a high-level governmental body with a membership which comprises the Minister for Health and the Commissioners of Health from the 36 Nigerian states and the Federal Capital Territory*. This activity was not done due to the lack of funding to support the process, since it was not an activity outlined in the PMV study protocol at inception coupled with the expiration of the timeline for the implementation of the study. This was a major limitation for the nation-wide adoption and scale-up of the initiative.

#### Development and strengthening of Health information system

According to the project protocol, PMVs were expected to submit reports of injectable service provision during the scheduled monitoring visits. They were trained on data management and record forms were provided to facilitate this as expressed below:

*As regards the record keeping, we were trained on how to keep the records of our clients and we must give them a copy of their next appointment date. Once our clients come, we check our book to be sure that it is truly her next appointment date (PMV _Oyo)*

The 6-month post-intervention evaluation revealed that only 50% of the PMVs had complete record of services provided. The reason cited for this sub-optimal record keeping practice was potential harassment from the law officials. During this current assessment, only a few PMVs had records for their services though they had the phone numbers of their clients and the next data of appointment for re-injection. According to them, most of the PMVs stopped keeping records of services, since the  pilot implementation research project was completed and they had been told to stop providing injectable contraceptive services though they continued to provide services due to demand from their clients.

## Discussion

This process evaluation study explored the implementation processes and elucidated the mechanisms through which a pilot implementation research project influenced the delivery of progestin-only injectable contraceptives through PMVs’ stores in three states in Nigeria*.* A huge population of women in developing countries have an unmet need for family planning [30]. Achieving global initiatives such as the Family Planning 2020 (FP2020) and the Sustainable Development Goals’ target of achieving universal access to family planning services [31] may remain a mirage if the potentials of the private sector are unexplored especially in densely populated countries. Drug shops which operate in the private sector are vital and ubiquitous outlets for the delivery of contraceptive services in low and middle-income countries [18, 30]. Unfortunately, the drug shops are underleveraged for the delivery of contraceptive services and this sector is least understood compared to family planning delivery in the public sector [18]. Our study has contributed to the body of knowledge and elucidated the implementation processes and causal mechanisms which influenced injectable contraceptive service provision by PMVs in a densely populated country and these are discussed subsequently.

### Training of human resources from the PMV stores

Training was a key activity which influenced the causal mechanism and outcomes of  the PMV pilot implementation research study. It remains a potent approach with equipping PMVs with the necessary information to provide safe injectable contraceptive services as reported in similar studies [32, 33]. PMVs can also learn from a standard curriculum, but it is not enough to ensure knowledge over time [26] and the study brought to fore the fact that knowledge outcomes do decrease after training as ascertained by the PMVs.

### Provision and utilisation of instructional materials and job aids

The use of job aids is as an effective tool to help providers remember and adhere to protocol for service provision [34]. Unfortunately, a low proportion of PMVs used the job aids. There are three possible reasons for this behavior. First, low usage may be due to concerns about violation of the law, because PMVs were no longer authorised to provide the services, since the previous project had ended. Second, PMVs may perceive that clients would not have confidence in their ability to provide the services if they use the job aids in their presence. Finally, it is possible that PMVs used the job aids during the first few weeks after training and might have gained mastery of the procedures with increased service provision. It is also likely that PMVs who have worked in Family Planning units have greater confidence in injectable contraceptive service provision than their counterparts without such experience. This issue requires further investigation. Nevertheless, future interventions must continue to emphasise the importance of use of aids to produce better outcomes [23]. In addition to training on the use of the job aids and the provision and distribution of different types/ranges [23], another strategy which may be explored in this population is the use of mobile phone job aids [35].

### Supply management chain and drug quality for injectable contraceptives

The supply of injectable contraceptives was a component of the intervention which had some gaps. Efforts were made to establish a reliable commodity supply mechanism by providing a seed stock of injectables to the PMVs and establishing re-supply linkages with credible organisations. However, there was a challenge sustaining the supply of Sayana Press in particular. This partly explains why more women reportedly used Depo-Provera and Noristerat than Sayana-Press. PMV shop is a business organisation created to make profit [35] and it is therefore understandable why the PMVs sourced the injectable contraceptives from the open market. Unfortunately, this practice is fraught with the risk of purchase of low-quality commodities.

### Injectable contraceptive service provision to clients

Among the current injectable contraceptive users, an overwhelming majority received their most recent injections from the same PMV as their previous injections. This might be due to the increased skills demonstrated by the PMVs in the areas of counselling and administration of injectables. The majority of the clients also reported that they got all the services they needed from the PMVs. This finding is consistent with previous findings on the reasons why client continue to patronize PMVs [36, 37, 38]. This may be linked to the intensive training that the PMVs got prior to the start of the project.

### Institutional mechanism to monitor and supervise injectable service provision

The monitoring activity provided an avenue for supportive supervision which ultimately boosted their confidence in delivering FP counselling, injectable contraceptives, and referral services as appropriate. In addition, the monitoring exercise gave them more recognition in the community as it portrayed government approval of the provision of injectable contraceptive services by PMVs. Our study thus supports the finding that rich and timely supervisory visits by the programme implementers and relevant government agencies to the PMVs is an important factor to the success of the intervention [39, 40].

### Referral linkages with private and public facilities

Overall, the level of referral is below expectation and requires improvement, Penn-kekanna and colleagues have reported similar findings [40]. Hebert and colleagues recommend that concrete interventions and efforts should be made to foster a more integrated and knowledgeable referral network for family planning services. This could be through network building, information-sharing [41, 42], distribution of guidelines with structured referral links and involvement of secondary level health workers in training primary-level healthcare workers [43] organisational network strengthening [44] and data capture in the national database on contraceptive service provision [20, 45].

## Conclusion

The PMVs continue to play important roles in FP service provision including injectable contraceptives. The PMVs deliver the services in response to the needs expressed by women. This reflects the weakness in the current health system which is unable to respond to women’s needs for timely access to contraception. Despite limitations reported on the pilot implementation research project, many of the clients expressed satisfaction and confidence in the ability of the PMVs to serve them and would recommend  them to their friends. This justifies the urgent need for the Nigerian government to implement the tiered PMV accreditation system to improve the overall quality of services rendered by the PMVs coupled with appropriate training, monitoring and regulation system. The accreditation system should serve as the mechanism through which the PMVs are integrated into the formal health care system. Furthermore, there is need to strengthen the referral system between the PMVs and other health professionals in both the private and the public health facilities.

## Supplementary Information

**Additional file 1.** Questionnaire for PMVs.

**Additional file 2.** Questionnaire for Female Client.

**Additional file 3.** Qualitative Interview Guides.

## Data Availability

The data sets are available upon request to the corresponding author. This can only be used for non-commercial purposes while maintaining participants’ confidentiality.

## Abbreviations

ARFH: Association for Reproductive and Family Health
BCS: Balanced counselling strategy
CHEW: Community health extension worker
CHO: Community health officer
CPR: Contraceptive prevalence rate
DHS: Demographic health survey
DKT: Deep K. Tyagi Foundation Nigeria
FGN: Federal government of Nigeria
FHI: Family Health International
FMoH: Federal Ministry of Health
FP: Family planning
IDI: In-depth interview
LGA: Local Government Area
MEC: Medical eligibility criteria
MRC: Medical Research Council
MMR: Maternal Mortality Ratio
NAPPMED: National Association for Proprietary and Patent Medicine Dealer
NDHS: Nigeria Demographic and Health Survey
NGO: Non-Governmental Organisation
NURHI: Nigerian Urban Reproductive Health Initiative
PATH: Program for Appropriate Technology in Health
PCN: Pharmacy Council of Nigeria
PM: Patent medicine
PMV: Patent Medicine Vendor
SFH: Society for family health
SMoH: State ministry of health
SPSS: Statistical Package for Social Sciences
SSA: Sub-Sahara Africa
ToC: Theory of change model
UNICEF: United Nations Children’s Fund
USAID: United States Agency for International Development
WHO: World Health Organisation
