# Supplementary material for: Process evaluation of an intervention to improve access to injectable contraceptive services through patent medicine vendors in Nigeria: a mixed methods study
Source: J Pharm Policy Pract. 2021 Nov 16;14(Suppl 1):88. doi: 10.1186/s40545-021-00336-5 (PMC8594092; doi:10.1186/s40545-021-00336-5)
Supplement: Supplementary file 1 — Additional file 1. Questionnaire for PMVs. [file 40545_2021_336_MOESM1_ESM.docx]

**Assessment of the Processes and Mechanisms of Influence of Proprietary Patent Medicine Vendors’ Training on Access to and Utilization of Injectable Contraceptives in Nigeria**

**Questionnaire for Trained PPMV**

| **IDENTIFICATION** | |
| --- | --- |
| STATE: _____________________________________________  LGA:  RESPONDENT CODE  Language of interview………………………………………………………… |  |

| INTERVIEW | | | | | | |
| --- | --- | --- | --- | --- | --- | --- |
| DATE |  | | | |  | |
| INTERVIEWER’S  NAME |  | | | | Interviewer's code | |
| INTERVIEW RESULT: (CIRCLE)  1 COMPLETED 4 PARTLY COMPLETED  2 POSTPONED 5 OTHER (SPECIFY)________________________  3 REFUSED | | | | | | |
| SUPERVISOR | | | DATA ENTERED BY: | | |  |
| NAME | |  | NAME |  | | |
| DATE | |  | DATE |  |  |  |

_________________________________ ______________

| **No.** | | **QUESTIONS AND FILTERS** | | **CODING CATEGORIES** | | **CODE** | | **SKIP** | |
| --- | --- | --- | --- | --- | --- | --- | --- | --- | --- |
|  | | **INT: Record the time of start of interview** | | Hour………….  Minutes…………. | |  | |  | |
|  | | Do you currently have the following commodities in stock?  **INT: Read out the following responses, circle if response is YES, multiple answers possible.** | | Depo-Provera (DMPA)  Noristerat (NET EN)  Sayana Press  Other______________________________ | | 01  02  03  04 | |  | |
|  | | At what price did you sell each vial for (not including separate fees charged for injecting the contraceptive)?  **INT: Read out the following responses, write responses as given** | | Depo-Provera (DMPA)  Noristerat (NET EN)  Sayana Press  Other_______________________________ | | _______  _______  _______  _______ | |  | |
|  | | In the past month, how many injections for each of the following medications did you give?  **INT: Read out the following responses, write responses as given** | | Depo Provera (DMPA)  Noristerat (NET EN)  Sayana Press | |  | |  | |
|  | | How much did you charge for each injection (including the cost for the vial of contraceptive and any fees for the injection)?  **INT: Read out the following responses, write responses as given** | | Depo-Provera (DMPA)  Noristerat (NET EN)  Sayana Press | | _______  _______  _______ | |  | |
|  | | In the past month, how many referrals to a health facility for administering injection did you make for the following medications?  **INT: Read out the following responses, write responses as given** | | Depo-Provera (DMPA)  Noristerat (NET EN)  Sayana Press | |  | |  | |
|  | | In the past month, how many clients returned to you with an adverse event/severe adverse event?  **INT: Write responses as given, clarify adverse event and severe adverse event** | | Number of clients that returned with adverse event  Number of clients that returned with severe adverse event  None | | 00 | | **109** | |
|  | | What were the adverse events that you received?  **INT: Do not probe, write response as given** | | Specify, ___________________  Specify, ___________________  Specify, ___________________ | | 01  02  03 | |  | |
|  | | What did you do when a client came back with adverse event?  **INT: Do not probe, write response as given, verbatim** | | Specify_____________________________ | |  | |  | |
|  | What are the common side-effects of injectable family planning methods?  **INT: Do not probe, response is spontaneous. Circle response, multiple answers possible** | None  Irregular bleeding (no pattern/regularity)  Heavy bleeding (regular timing, heavier during cycle)  Prolonged bleeding (longer bleeding during cycle)  Infrequent or absence of bleeding (long duration between menstruations)  Weight gain  Headaches  Dizziness  Nausea  Breast tenderness  Mood change  Decrease in sex drive  Delayed return to fertility  Blurred vision  Acne/pimples  Hair loss  Temporary mild/moderate skin irritation  Don’t know  Others (specify)______________________  Others (specify)______________________  Others (specify)_______________________ | | 00  01  02  03  04  05  06  07  08  09  10  11  12  13  14  15  16  17  18  19  20 | |  | |  |  |
|  | Can injectable family planning methods prevent HIV/STIs?  **INT: Circle response, do not leave blank** | Yes  No  Don’t know | | 01  00  02 | |  | |  |  |
|  | Under what health conditions should a woman **NOT** be provided progestin-only family planning injectable (Depo-Provera/Noristerat)?  **INT: Do not probe, response is spontaneous. Circle response, multiple answers possible** | Have acute deep vein thrombosis  Have liver tumour (tumour/hepatitis or jaundice-yellow skin or eyes)  Are breastfeeding up to 4 weeks postpartum  Have blood pressure 160/100 mmHG & above  Have diabetes (high sugar in blood) with vascular complications  Have unexplained vaginal bleeding (between menstrual periods or after intercourse)  Have multiple risk factors for cardiovascular disease  Have current/history of stroke or ischaemic heart disease (constant sustained pain in calf/back of lower leg  Have noticed their migraines with auro getting worse while taking progestin-only injectables  Have rheumatic disease such as lupus (conditions: joint swelling, stiffness, and/or pain that comes and goes, deformity at joint)  Have a history of breast cancer (lump in breast) & no evidence of current disease for 5 years  Don’t know  Others (specify)_____________­­­­­­­__________  Others (specify)_______________________ | | 01  02  03  04  05  06  07  08  09  10  11  12  13  14 | |  | |  |  |
|  | When can a woman start progestin-only (Depo-Provera/Noristerat) injectable family planning method?  **INT: Do not probe, response is spontaneous. Circle response, multiple answers possible** | When she is not pregnant  Within the 1^st^ 7 days of a menstrual cycle without back-up method  After the 1^st^ 7 days of a menstrual cycle then provide back-up method for 1^st^ 7 days after injection  Four weeks after childbirth  Immediately after a miscarriage or abortion  Immediately after stopping another method  Don’t know  Other (specify)________________________  Other (specify)________________________ | | 01  02  03  04  05  06  07  08  09 | |  | |  |  |
|  | What counselling messages should be given to someone who is being provided progestin-only injectable contraceptives?  **INT: Do not probe, response is spontaneous. Circle response, multiple answers possible** | Injectable does not protect against STI/HIV and condom should be used if partners are at risk  Do not massage the injection site  Skin may be irritated at the injection site for several days  Any problems with very heavy bleeding, seek services from provider  Return for next injection in 2 (NET) or 3 (DMPA) months  Other (specify) _______________________  Other (specify) _______________________ | | 01  02  03  04  05  06  07 | |  | |  |  |
|  | Please describe steps to be taken when selling injectable family planning methods only (not administering).  **INT: Do not probe, response is spontaneous. Circle response, multiple answers possible** | Ask about previous use of the method  Counsel on advantages & disadvantages  Counsel on side effects  Check expiration of vial  Provide clean syringes & needles  Provide alcohol swabs  Provide health facility referral information  Complete client intake card  Other (specify)________________________  Other (specify)________________________  Other (specify)________________________  Other (specify)________________________  Other (specify)________________________ | | 01  02  03  04  05  06  07  08  09  10  11  12  13 | |  | |  |  |
|  | Which family planning methods do you sell at your PM shop?  **INT: Read out the following responses, circle if response is YES, multiple answers possible** | None  Pill  IUD/Loop  Injectables  Implant  Male Condom  Female Condom  Diaphragm  Spermicide  Cycle beads  Other (specify) _____________  Other (specify) _____________ | | 00  01  02  03  04  05  06  07  08  09  10  11 | |  | |  |  |
|  | Have you experienced any stock outs of family planning methods in the past 30 days (a situation when clients request for a drug and you do not have in the shop)?  **INT: Read out the following responses, circle if response is YES, multiple answers possible only** | Injectable Contraceptive  Pills  Condoms  Spermicide  Emergency contraceptive  IUD  Diaphragm  Standard Days Method (Cycle Beads)  Implant  None | | 01  02  03  04  05  06  07  08  09  00 | | **119**  **119**  **119**  **119**  **119**  **119**  **119**  **119**  **119** | |  |  |
|  | What type of injectable family planning method have you been stocked out of in the past 30 days?  **INT: Read out the following responses, circle if response is YES, multiple answers possible only** | Depo-provera, DMPA  Noristerat, NET-EN  Sayana Press | | 01  02  03 | |  | |  |  |
|  | What other types of injections have you administered in your shop?  **INT: Do not probe, record response as given** | Specify____________________________  Specify____________________________ | |  | |  | |  |  |
|  | What supplies or equipment are required when administering injectable family planning methods? Please mention all materials required.  **INT: Do not probe, response is spontaneous. Circle response, multiple answers possible** | Safety box  Syringe  Needles  Tray or kidney dish  Cotton swab  Methylated spirit or clean water  Soap  Uniject  Clean running water  Don’t know  Other (specify) _______________________  Other (specify) _______________________  Other (specify) _______________________  Other (specify) _______________________ | | 01  02  03  04  05  06  07  08  09  10  11  12  13  14 | |  | |  |  |
|  | What instructions should be provided to women after administering the injection?  **INT: Do not probe, response is spontaneous. Circle response, multiple answers possible** | Do not rub injection site  Provide information about possible side effects  When to return for next injection  Refer clients to health facility if they experience:  Suspicion about pregnancy  Concerns/questions about the method  Any significant changes in health  No instructions given  Other (specify) _______________________  Other (specify) _______________________  Other (specify) _______________________  Other (specify) _______________________ | | 01  02  03  04  05  06  07  08  09  10  11 | |  | |  |  |
|  | Apart from the training on injectable contraceptives by Population Council, have you participated in anyother learning or training events on family planning?  **INT: Circle response, do not leave blank** | Yes  No | | 01  00 | |  | |  |  |
|  | Which person or organization organized the training? |  | |  | |  | |  |  |
|  | Have you been visited by person or organization coming to sell you injectable contraceptive supplies or products?  **INT: Circle response, do not leave blank** | Yes  No | | 01  00 | |  | |  |  |
| **General Knowledge of Depo-Provera Provision**  **INT: “Now I would like to ask you some questions about a specific type of injectable family planning method called Depo-Provera.”** | | | | | | | | |  |
|  | What type of injection device is used to administer Depo-Provera?  **INT: Do not probe, response is spontaneous. Circle response, one answer possible** | Syringe and needle (IM)  Uniject device (subcutaneous)  Don’t know  Other (specify)____________ | | 01  02  03  04 | |  | |  |  |
| Key, | Please describe the steps that should be taken when administering Depo-Provera.  **INT: Do not probe, response is spontaneous. Circle response, multiple answers possible** | Check label carefully for expiration date  Ask client for her preferred injection location  Mix contents by rocking vial back and forth    Wash hands  Pierce top of vial with sterile needle  Fill syringe  Expel air from syringe  Clean injection site with cotton soaked in methylated spirit or water  Inject drug slowly  Apply pressure on injection site with cotton wool  Do not rub injection site  Dispose needle & syringe in hazard waste container  Record all information on client’s card  Don’t know  Other (specify) _______________________  Other (specify) _______________________  Other (specify) _______________________  Other (specify) _______________________ | | 01  02  03  04  05  06  07  08  09  10  11  12  13  14  15  16  17  18 | |  | |  |  |
|  | Where can Depo-Provera be given on the body?  **INT: Do not probe, response is spontaneous. Circle response, multiple answers possible** | Upper arm (deltoid)  Buttocks  Don’t know  Other (specify)________________ | | 01  02  03  04 | |  | |  |  |
|  | When should a client be told to return after receiving Depo-Provera?  **INT: Do not probe, response is spontaneous. Circle response, One answer possible** | Every month  Every two months (8 weeks)  Every three months (13 weeks)  Don’t know  Other (specify) _______________________  Other (specify) _______________________ | | 01  02  03  04  05  06 | |  | |  |  |
|  | How should Depo-Provera be stored?  **INT: Do not probe, response is spontaneous. Circle response, multiple answers possible** | Must be stored at room temperature  Away from direct sunlight/heat  In the refrigerator  Don’t know  Other (specify)________________________  Other (specify)________________________ | | 01  02  03  04  05  06 | |  | |  |  |
|  | What are the safety precautions to employ when administering Depo-Provera?  **INT: Do not probe, response is spontaneous. Circle response, multiple answers possible** | Wash hands with soap and running water  Handle needles safely  Check expiry date on Depo-Provera vial  Don’t know  Other (specify)_______________________  Other (specify)_______________________ | | 01  02  03  04  05  06 | |  | |  |  |
| **General Knowledge of Noristerat Provision**  **INT: “Now I would like to ask you some questions about a specific type of injectable family planning method called Noristerat”** | | | | | | | | |  |
|  | What type of injection device is used to administer Noristerat?  **INT: Do not probe, response is spontaneous. Circle response, one answer possible** | Syringe and needle (IM)  Uniject device (subcutaneous)  Don’t know  Other (specify)____________ | | 01  02  03  04 | |  | |  |  |
|  | Please describe the steps that should be taken when administering Noristerat.  **INT: Do not probe, response is spontaneous. Circle response, multiple answers possible.** | Check label carefully for expiration date  Ask client for her preferred injection site  Mix contents by rub vial in-between the palm  to enhance withdrawal of the oily content  Wash hands  Pierce top of vial with sterile needle  Fill syringe  Expel air from syringe  Clean injection site with cotton soaked in methylated spirit or water  Aspirate needle to ensure there is no blood  Inject drug slowly  Apply pressure on injection site with cotton wool  Dispose needle & syringe in hazard waste container  Record all information on client’s card  Don’t know  Other (specify) _______________________  Other (specify) _______________________  Other (specify) _______________________  Other (specify) _______________________ | | 01  02  03  04  05  06  07  08  09  10  11  12  13  14  15  16  17  18 | |  | |  |  |
|  | Where can Noristerat be given on the body?  **INT: Do not probe, response is spontaneous. Circle response, multiple answers possible.** | Upper arm (deltoid)  Buttocks  Don’t know  Other (specify)________________ | | 01  02  03  04 | |  | |  |  |
|  | When should a client be told to return after receiving Noristerat?  **INT: Do not probe, response is spontaneous. Circle response, One answer possible** | Every month  Every two months (8 weeks)  Every three months (13 weeks)  Don’t know  Other (specify) _______________________  Other (specify) _______________________ | | 01  02  03  04  05  06 | |  | |  |  |
|  | How should Noristerat be stored?  **INT: Do not probe, response is spontaneous. Circle response, multiple answers possible.** | Must be stored at room temperature  Away from direct sunlight/heat  In the refrigerator  Don’t know  Other (specify) _______________________  Other (specify) _______________________ | | 01  02  03  04  05  06 | |  | |  |  |
|  | What are the safety precautions to employ when administering Noristerat?  **INT: Do not probe, response is spontaneous. Circle response, multiple answers possible.** | Wash hands with soap and running water  Handle needles safely  Check expiry date on Noristerat vial  Don’t know  Other (specify) _______________________  Other (specify) _______________________ | | 01  02  03  04  05  06 | |  | |  |  |
| **General Knowledge of Sayana Press Provision**  **INT: “Now I would like to ask you some questions about a specific type of injectable family planning method called Sayana Press.”** | | | | | | | | |  |
|  | What type of injection device is used to administer Sayana Press?  **INT: Do not probe, response is spontaneous. Circle response, One answer possible** | Syringe and needle (IM)  Uniject device (subcutaneous)  Don’t know  Other (specify)____________ | | 01  02  03  04 | |  | |  |  |
|  | Please describe the steps that should be taken when administering Sayana Press.  **INT: Do not probe, response is spontaneous. Circle response, multiple answers possible.** | Wash hands  Ask client preferred injection site  Clean injection site with cotton soaked in methylated spirit or water  Check expiration date on the pouch  Check Sayana is at room temperature  Hold Uniject by the port and shake vigorously for 30 seconds  Activate the Uniject  Pinch the skin of injections site  Insert straight into skin at a slight downward angle  Squeezes reservoir slowly to inject (5-7 secs)  Dispose uniject in safety box  Record all information on client’s card  Don’t know  Other (specify) _______________________  Other (specify) _______________________  Other (specify) _______________________  Other (specify) _______________________ | | 01  02  03  04  05  06  07  08  09  10  11  12  13  14  15  16  17 | |  | |  |  |
|  | Where can Sayana Press be given on the body?  **INT: Do not probe, response is spontaneous. Circle response, multiple answers possible** | Back of the upper arm  In the abdomen  On the front of the thigh  Any part of the body  Don’t know  Other (specify)_______________________  Other (specify)_______________________ | | 01  02  03  04  05  06  07 | |  | |  |  |
|  | When should a client be told to return after receiving Sayana Press?  **INT: Do not probe, response is spontaneous. Circle response, one answers possible.** | Every month  Every two months (8 weeks)  Every three months (13 weeks)  Don’t know  Other (specify) _______________________  Other (specify) _______________________ | | 01  02  03  04  05  06 | |  | |  |  |
|  | How should Sayana Press be stored?  **INT: Do not probe, response is spontaneous. Circle response, multiple answers possible** | Must be stored at room temperature  Away from direct sunlight/heat  In the refrigerator  Don’t know  Other (specify)_______________________  Other (specify)_______________________ | | 01  02  03  04  05  06 | |  | |  |  |
|  | What are the safety precautions to employ when administering Sayana Press?  **INT: Do not probe, response is spontaneous. Circle response, multiple answers possible** | Wash hands with soap and running water  Check expiry date on uniject device  Don’t know  Other (specify)___________________ | | 01  02  03  04 | |  | |  |  |
| General Knowledge of Safety Precautions and Infection Prevention  **INT: “Now I would like to ask you some questions about Safety Precautions and Infection Prevention”** | | | | | | | | |  |
|  | Can you please tell me what are the ways to handle needles/syringes safely?  **INT: Do not probe, response is spontaneous. Circle response, multiple answers possible.** | Do not touch the needles  Do not recap the needles  Discard the needle immediately after use  Ensure that any sharp is disposed of in the sharp box  Do not overfill the sharp box  Don’t know  Other (specify)________________ | | 01  02  03  04  05  06  07 | |  | |  |  |
|  | What precautions should be taken to prevent infection from a needle stick injury?  **INT: Do not probe, response is spontaneous. Circle response, multiple answers possible.** | Wash hands with soap and water immediately before and after giving injection  Use box for disposal of sharps and needles  Handle needles carefully  Don’t know  Other (specify)______________ | | 01  02  03  04  05 | |  | |  |  |
|  | What should be done in the case of a needle stick injury?  **INT: Do not probe, response is spontaneous. Circle response, multiple answers possible.** | Wash the wound site with soap and water immediately  Ensure nothing is put on wound site after cleaning it  Apply a plaster or bandage to wound site  Don’t know  Other (specify)_________________ | | 01  02  03  04  05 | |  | |  |  |
| **INT: Now I would like to ask you a few questions about your experience as a participant in this study.** | | | | | | | |  |  |
|  | I am going to read out several statements and would like to know if you strongly agree, agree, disagree or strongly disagree with it.  “The training improved my knowledge and skills in providing injectable contraceptive services” | Strongly agree  Agree  Disagree  Strongly disagree | | 1  2  3  4 | |  | |  |  |
|  | Do you agree strongly agree, agree, disagree or strongly disagree with the following statement?  “The training improved my knowledge and skills in FP counselling” | Strongly agree  Agree  Disagree  Strongly disagree | | 1  2  3  4 | |  | |  |  |
|  | On a scale of 1 is very easy and 5 is very difficult, how easy or difficult did you find the training that you participated in as part of this study? | Very easy  Easy  Moderate  Difficult  Very difficult | | 1  2  3  4  5 | | **149**  **149**  **149** | |  |  |
|  | (INT) If response to question 151 is Difficult or Very difficult, ask:  What aspect of the training did you find difficult? |  | |  | |  | |  |  |
|  | Do you strongly agree, agree, disagree or strongly disagree with the following statement?  “I benefitted from the monitoring visits” | Strongly agree  Agree  Disagree  Strongly disagree | | 1  2  3  4 | |  | |  |  |
|  | List three ways we can improve monitoring visits. | 1._______________________________  2._______________________________  3._______________________________ | |  | |  | |  |  |
|  | How often do you use the job aides when providing family planning services? Do you use them with every FP client, most of FP clients, some FP clients or no FP clients? | No FP clients  Some FP clients  Most FP client  All FP clients | | 1  2  3  4 | | **153** | |  |  |
|  | Please tell me all the job aides that you use when providing FP services | BCS+ Cards  MEC Wheel  FHI Checklist for screening clients who want to initiate DMPA (or NET-EN)  FHI Reinjection job aid  Other___________________________ | | 01  02  03  04  05 | |  | |  |  |
|  | As a PPMV, do you interact or coordinate with the health facilities in your LGA? | Yes  No | | 1  2 | | **154**  **1555** | |  |  |
|  | (INT) If response is Yes to question 153, ask:  Describe how you interact/coordinate with the health facilities in your LGA? |  | |  | | **156** | |  |  |
|  | (INT) If response is No to question 153, ask:  What is the main reason you do not interact/coordinate with the health facilities? |  | |  | |  | |  |  |
|  | During the course of the Population council funded study , name the top three things that helped you the most in providing injectable contraceptive services? | Linkages of PPMVs by Population council with organizations to procure injectable contraceptives     1. Population Council’s support in facilitating the required legal and regulatory changes to enable drugshops to provide injectable services. 2. Monitoring of injectable service provision by government agencies and Population council 3. Training of PPMVs on injectable service provision. 4. PPMVs motivations in provision of health services. 5. Increased access to services among young people through drugshops. 6. Development of referral chains with the public system or formal private providers. 7. Development and strengthening of health management information systems (data and records) 8. Educating users about appropriate use of injectables and possible side effects 9. Getting stakeholders, particularly formal providers within the publics system to engage with drug shops.   _____________________________ | |  | |  | |  |  |
| 161 | Record the time of end of interview | Hour………….  Minutes…………. | |  | |  | |  |  |

**We thank you for taking the time to answer all our questions.**
