# Supplementary material for: Process evaluation of an intervention to improve access to injectable contraceptive services through patent medicine vendors in Nigeria: a mixed methods study
Source: J Pharm Policy Pract. 2021 Nov 16;14(Suppl 1):88. doi: 10.1186/s40545-021-00336-5 (PMC8594092; doi:10.1186/s40545-021-00336-5)
Supplement: Supplementary file 2 — Additional file 2. Questionnaire for Female Client. [file 40545_2021_336_MOESM2_ESM.docx]

**Assessment of the Processes and Mechanisms of Influence of Proprietary Patent Medicine Vendors’ Training on Access to and Utilization of Injectable Contraceptives in Nigeria**

**CLIENT INTERVIEW QUESTIONNAIRE**

| **IDENTIFICATION** | |
| --- | --- |
| STATE:  LGA:  PMS SHOP LOCATION  RESPONDENT CODE  Interview language_________________________________________ |  |

| INTERVIEW | | | | | | |
| --- | --- | --- | --- | --- | --- | --- |
| DATE |  | | | | Day Mont Year | |
| INTERVIEWER’S  NAME |  | | | | Interviewer's code | |
| INTERVIEW RESULT: (CIRCLE)  1 COMPLETED 4 PARTLY COMPLETED  2 POSTPOND 5 OTHER (SPECIFY)________________________  3 REFUSED | | | | | | |
| SUPERVISOR | | | DATA ENTERED BY: | | |  |
| NAME | |  | NAME |  | | |
| DATE | |  | DATE |  |  |  |

1. Age in years ……………………………………………………………………………………...
2. Educational Qualification (a) No formal education. (b) Primary education (c) Secondary Education. (d) Tertiary education
3. Marital status. (a) Single. (b) Married/living with partner. (c) Divorced. (d) Widowed
4. Religion (a) Christianity. (b)Islam. (c) Traditional religion (d) Others. (specify)
5. Ethnicity. (a) Yoruba (b) Igbo (c)Hausa (d) Others. (specify)

| **No.** | **QUESTIONS AND FILTERS** | **CODING CATEGORIES** | | | | **CODE** | | **SKIP** | |  |
| --- | --- | --- | --- | --- | --- | --- | --- | --- | --- | --- |
|  | **INT: Record the time.** | Hour………….  Minutes…………. | | | |  | |  | |  |
| **Experience with injectable family planning services** | | | | | | | | | |  |
|  | Are you currently using an injectable family planning method? (For example, Sayana Press, DMPA, Noristerat, Mesigyna, Cyclofem?)  **INT: Circle response, do not leave blank** | | Yes  No | | | | 01  00 | | **104** | |
|  | Have you received your most recent injection?  **INT: Circle response, do not leave blank** | | Yes  No | | | | 01  00 | | **113** | |
|  | Why are you not currently using an injectable contraceptive?  **INT: Read out the following responses, circle if response is YES, only one answer possible** | | Haven’t gone back yet, forgot    Haven’t gone back yet, didn’t have time  Do not like the injectable contraceptive method  Stopped method because of side effects  Stopped method because want to become pregnant (**thank client, end interview)**  Stopped method because I am pregnant  (**thank client, end interview)**  Stopped method because of my partner  Stopped method because PPMVno longer providesservices  (**thank client, end interview)**  **Other (Specify)______________________** | | | | 01  02  03    04  05  06  07  07 | | **106**  **107**  **147**  **147**  **147**  **147** | |
|  | What did you not like about the injectable contraceptive?  **INT: Do not probe, write response as given** | | Specify____________________ | | | |  | |  | |
|  | What side effects did you experience?  **INT: Do not probe, response is spontaneous. Circle response, multiple answers possible** | | Irregular bleeding (no pattern/regularity)  Heavy bleeding (regular timing, heavier during cycle)  Prolonged bleeding (longer bleeding during cycle)  Infrequent or absence of bleeding (long duration between menstruations)  Weight gain  Headaches  Dizziness  Nausea  Breast tenderness  Mood change  Decrease in sex drive  Delayed return to fertility  Blurred vision  Acne/pimples  Hair loss  Temporary mild/moderate skin irritation  Others (specify)______________________  Others (specify)______________________  Others (specify)_______________________ | | | | 01  02  03  04  05  06  07  08  09  10  11  12  13  14  15  16  17  18  19 | |  | |
|  | Are you using a different family planning method?  **INT: Circle response, do not leave blank** | | Yes  No | | | | 01  00 | | **110** | |
|  | Why are you not currently using a method?  **INT: Do not probe, write response as given**  (**thank client and end interview)** | | Specify______________________________  (**thank client, end interview)** | | | |  | | **147** | |
|  | Which method are you currently using?  **INT: Do not probe, response is spontaneous. Circle response, one answer possible** | | Female sterilization  Male sterilization  Pill  IUD  Injectables (e.g. Sayana Press, DMPA, Noristerat, Mesigyna, Cyclofem)  Implants  Male Condom  Female Condom  Diaphragm  Spermicide  Rhythm Method  Withdrawal  Standard Days Method (SDM)  Other (Specify)_______________________ | | | | 01  02  03  04  05  06  07  08  09  10  11  12  13  14 | | **113** | |
|  | Where did you receive that family planning method?  **INT: Read out the following responses, circle if response is YES, only one answer possible** | | Same PPMV  Different PPMV  Private health facility  Public health facility  Friend  Other(specify)______________________ | | | | 01  02  03  04  05  06 | |  | |
|  | Why did you go there for contraceptive services?  **INT: Do not probe, write response as given**  (**thank client and end interview)** | | Other(specify)______________________  (**thank client, end interview)** | | | |  | | **147** | |
| **Continuing Injectable Contraceptive Use Survey** | | | | | | | | | | |
|  | What type of injectable family planning method are you currently using?    **INT: Read out the following responses, circle if response is YES, only one answer possible** | | Depo-Provera  Noristerat, NET-EN  Mesigyna, Norigy  Cyclofem  Sayana Press  Don’t know  Other (specify)_______________ | | | | 01  02  03  04  05  06  07 | |  | |
|  | How long have you been using this injectable?  **INT: Do not probe, write response as given** | | Days  Months  Years | | | |  | |  | |
|  | Where did you receive your most recent injectable contraceptive injection?  **INT: Do not probe, response is spontaneous. Circle response, one answer possible** | | Same PPMV as last injection  Different PPMV from last injection  Public health facility  Private health facility  CHEW  Other(specify)________________________ | | | | 01  02  03  04  05  06 | | **117**  **117** | |
|  | Why did you choose to go to this provider for your injectable contraceptive?  **INT: Do not probe, response is spontaneous. Circle response, multiple answers possible.**  **(thank the client and end the interview)** | | More convenient location  More convenient hours  Price is lower  Quality of services is better  I needed other services from the same location  Other(specify)____________________ | | | | 01  02  03  04  05  06 | | **147**  **147**  **147**  **147**  **147**  **147** | |
|  | What are your reasons for going to this PM shop to receive injectable services instead of going elsewhere?  **INT: Do not probe, response is spontaneous. Circle response, multiple answers possible** | | Hours of operation  Convenient location  Lower cost  No waiting time  Anonymous care  PPMVs shop always have the drug  Receive other services from this PPMV  Know the PPMV  Other(specify)___________________________________________________________ | | | | 01  02  03  04  05  06  07  08  09 | |  | |
|  | What injectable family planning services did you receive from the PPMV at your most recent visit?  **INT: Read out the following responses, circle if response is YES, only one answer possible** | | Purchased injectable, did not receive injection  Purchased injectable and referred to facility for injection  Purchased and received injectable shot  Referred to health facility for injectable  Counselled on injectable methods, no purchase  Other (specify)________________________ | | | | 01  02  03  04  05  06 | | **147**  **147**  **147**  **147** | |
|  | Did the PPMV ask on which part of the body you prefer to take the injection?  **INT: Circle response, do not leave blank** | | Yes  No | | | | 01  00 | |  | |
|  | Which part of your body did the PPMV inject the shot?  **INT: Read out the following responses, circle if response is YES, only one answer possible** | | Arm (deltoid muscle)  Hip  Buttocks  Anterior thigh (front of thigh)  Abdomen  Back of arm  Other(specify)________________________  ___________________________________ | | | | 01  02  03  04  05  06  07 | |  | |
|  | How much did you pay for all injectable services received from the PPMV?  **INT: Do not probe, write response as given** | | Cost in Naira | | | |  | |  | |
|  | How easy was it for you to cover the costs of the injectable contraceptive services received from the PMV?  **INT: Read out the following responses, circle if response is YES, only one answer possible** | | Very easy  Easy  Neutral  Difficult  Very difficult | | | | 01  02  03  04  05 | |  | |
|  | How comfortable did you feel asking PPMVs questions at your last visit?  **INT: Read out the following responses, circle if response is YES, only one answer possible** | | Very comfortable  Somewhat comfortable  Neutral  Somewhat uncomfortable  Very uncomfortable | | | | 01  02  03  04  05 | |  | |
|  | How confident do you feel that the PPMV will keep your information private?  **INT: Read out the following responses, circle if response is YES, only one answer possible** | | Very confident  Somewhat confident  Neutral  Somewhat unconfident  Very unconfident | | | | 01  02  03  04  05 | |  | |
|  | Did you feel that the PPMV was knowledgeable about injectable contraceptives?  **INT: Read out the following responses, circle if response is YES, only one answer possible** | | Very knowledgeable  Somewhat knowledgeable  Neutral  Somewhat unknowledgeable  Very unkowledageble | | | | 01  02  03  04  05 | |  | |
|  | During your family planning consultation did the PPMV say or do any of the following? **(INT: Ask each of the items below and record response)** | | **Yes** | **No** | **Can’t recall** | |  | |  | |
| a. | Ask the reason for your visit? | | 1 | 0 | 2 | |  | |  | |
| b. | Ask when your last menstruation was? | | 1 | 0 | 2 | |  | |  | |
| c. | Provide you information about different family planning methods? | | 1 | 0 | 2 | |  | |  | |
| d. | Reason why you want to use family planning injectable | | 1 | 0 | 2 | |  | |  | |
| e. | Ask whether you had taken injectable family planning method in the past? | | 1 | 0 | 2 | |  | |  | |
| f. | Talk about possible side effects of the injectable? | | 1 | 0 | 2 | |  | |  | |
| g. | Suggest any action(s) to resolve the problem with the side effect? | | 1 | 0 | 2 | |  | |  | |
| h. | Tell you what to do if you have any problems with the injectable? | | 1 | 0 | 2 | |  | |  | |
| i. | Tell you when to return for follow-up? | | 1 | 0 | 2 | |  | |  | |
|  | What did the PPMV tell you to do if you have any problems with the injectable besides normal side effects?  **INT: Do not probe, response is spontaneous. Circle response, one answer possible** | | To visit a health facility  To return to the PPMV  Other (specify)________________________  ___________________________________ | | | | 01  02  03 | |  | |
|  | What was the outcome of this visit to the PPMV? Did you decide to:  **INT: Read out the following responses, circle if response is YES, only one answer possible** | | Adopt a method  Continue with same method  Switch contraceptive method  Switch between injectable method  Stop using method (due to problems)  Stop using method (elective-no problems)  Others (specify)______________________ | | | | 01  02  03  04  05  06  07 | |  | |
|  | Did you get all the services you needed from the PPMV?  **INT: Circle response, do not leave blank** | | Yes  No | | | | 01  00 | | **131** | |
|  | What additional services did you need?  **INT: Do not probe, write response as given** | | Specify______________________________  ___________________________________ | | | |  | |  | |
|  | Will you return to this this PMV shop for your next injection?  **INT: Circle response, do not leave blank** | | Yes  No | | | | 01  00 | | **133** | |
|  | Where will you go for your next injection?  **INT: Do not probe, response is spontaneous. Circle response, one answer possible** | | Different PPMV  Government Health Facility  Don’t Know  Other________________ | | | | 01  02  03  04 | |  | |
|  | How satisfied were you with the services you received?  **INT: Read out the following responses, circle if response is YES, only one answer possible** | | Satisfied  Dissatisfied | | | | 01  02 | | **134**  **135** | |
|  | Reasons for satisfcation | | Specify ____________________________ | | | |  | |  | |
|  | Reasons for dissatisfaction | | Specify ____________________________ | | | |  | |  | |
|  | How many times have you received injectable services from this PPMV since you started using the injectable method?  **INT: Do not probe, write response as given** | | No. of visits to PPMV | | | |  | |  | |
|  | Did you experience any side effects in the last three months?  **INT: Circle response, do not leave blank** | | Yes  No | | | | 01  00 | | **147** | |
|  | What side effects did you experience?  **INT: Do not probe, response is spontaneous. Circle response, multiple answers possible** | | None  Irregular bleeding (no pattern/regularity)  Heavy bleeding (regular timing, heavier during cycle)  Prolonged bleeding (longer bleeding during cycle)  Infrequent or absence of bleeding (long duration between menstruations)  Weight gain  Headaches  Dizziness  Nausea  Breast tenderness  Mood change  Decrease in sex drive  Delayed return to fertility  Blurred vision  Acne/pimples  Hair loss  Temporary mild/moderate skin irritation  Others (specify)______________________  Others (specify)______________________  Others (specify)_______________________ | | | | 00  01  02  03  04  05  06  07  08  09  10  11  12  13  14  15  16  17  18  19 | |  | |
|  | What decisions will you make to continue using family planning due to these side effect(s)?  **INT: Do not probe, response is spontaneous. Circle response, one answer possible** | | It is manageable and I am going to continue to use the method  I am going to discontinue the method because of this side effect  This side effect occurred because I receive the injection from PPMV. It will not have occurred if injection were given in clinic or hospital  I have this effect because PPMVs usually provide sub-standard product  Any other perception (specify)---------------------------------------------------------------------------- | | | | 01  02  03  04  05 | |  | |
|  | Have you reported this/these side effect(s) to the PPMV who provided you the injectable?  **INT: Circle response, do not leave blank** | | Yes  No | | | | 01  00 | | **141** | |
|  | What assistance or advice was given to you to address this side effect?  **INT: Do not probe, response is spontaneous Circle response, one answer possible** | | No advice or suggestions given  To discontinue the method altogether  To discontinue the method until side effects, stop and then continue again  Other (specify)_________________________ | | | | 01  02  03  04 | |  | |
|  | Have you reported this side effect to another health care provider?    **INT: Circle response, do not leave blank** | | Yes  No | | | | 01  00 | | **144** | |
|  | Which health care provider did you report it to?  **INT: Do not probe, response is spontaneous. Circle response, one answer possible** | | Physician  Pharmacist  CHEW  Other (specify)__________________ | | | | 01  02  03  04 | |  | |
|  | What assistance or advice was given to you to address this side effect?  **INT: Do not probe, response is spontaneous Circle response, one answer possible** | | No advice or suggestions given  To discontinue the method altogether  To discontinue the method until side effects, stop and then continue again  Other (specify)_________________________ | | | | 01  02  03  04 | |  | |
|  | Where else have you gone for injectable services in the last three months?  **INT: Do not probe, response is spontaneous. Circle response, one answer possible** | | Nowhere, only been to this PM shop  Another PM shops  Health facility  CHEW  Pharmacy  Relative  Other (specify)________________________ | | | | 01  02  03  04  05  06  07 | |  | |
|  | What do you like about the services you have received from the PPMV you recently visited for injectable contraceptive services?  **INT: Do not probe, response is spontaneous. Circle responses, multiple answer possible** | | Nothing  PPMV is very knowledgeable  Cost of the injectable services  Hours of operation are convenient  This PPMV is a friend/relative/neighbour  Location of PPMV is convenient  Store always has the drugs in stock  I received all the information I needed on injectable contraceptives  __________________________________  .__________________________________  .__________________________________ | | | | 00  01  02  03  04  05  06  07  08  09  10 | |  | |
|  | Tell me what you disliked about the injectable services you received?  **INT: Do not probe, response is spontaneous. Circle responses, multiple answer possible** | | Nothing  The PPMV requested my phone number  PPMV does not have enough drugs available  Shop hours are inconvenient  Cost of injectable services  Side effects related to the injectable contraceptives  1.__________________________________  2.__________________________________  3.__________________________________ | | | | 00  01  02  03  04  05  06  07  08 | |  | |
|  | Based on your most recent experience, would you recommend this PPMV to someone else who needs injectable services?  **INT: Circle response, do not leave blank** | | Yes  No | | | | 01  00 | |  | |
|  | Where do you intend to go for your next injectable services?  **INT: Do not probe, response is spontaneous. Circle response, one answer possible** | | The same PM shops  Another PM shops  Health facility  CHEW  Pharmacy  Other specify_____________________ | | | | 01  02  03  04  05  06 | |  | |
|  | Why will you go there?  **INT: Do not probe, response is spontaneous. Circle response, one answer possible** | | PPMV is very knowledgeable  Satisfied with the services  I go to this PMV for other health services  This PMV is a friend/relative/neighbour  Comfortable with this PPMV  His/her shop is clean/orderly  The shop is in a convenient location  Other____________________________ | | | | 01  02  03  04  05  06  07  08 | |  | |
|  | Record the time and thank the respondent. | | Hour………….  Minutes…………. | | | |  | |  | |
