# Supplementary material for: Process evaluation of an intervention to improve access to injectable contraceptive services through patent medicine vendors in Nigeria: a mixed methods study
Source: J Pharm Policy Pract. 2021 Nov 16;14(Suppl 1):88. doi: 10.1186/s40545-021-00336-5 (PMC8594092; doi:10.1186/s40545-021-00336-5)
Supplement: Supplementary file 3 — Additional file 3. Qualitative Interview Guides. [file 40545_2021_336_MOESM3_ESM.docx]

**Assessment of the Processes and Mechanisms of Influence of Proprietary Patent Medicine Vendors’ Training on Access to and Utilization of Injectable Contraceptives in Nigeria**

**In-depth interview guide for PPMVs**

**Note to Interviewer: *Share the study information sheet and request for signed consent***

**Demographics, TAKE NOTES** (identifying information to be kept separate from interview transcripts)

Interview #___________________

**Background Information**

1. Gender
2. Age at last birthday
3. Highest educational qualification
4. Religion
5. Participant’s organization and contact details (fill in beforehand if possible)
6. Year of establishment of the PPMV store

**REQUEST TO TURN ON RECORDERS AT THIS POINT IN THE INTERVIEW**

**Implementation Evaluation**

1. In your own words, describe the activities implemented on this study. *What happened? general opinion and views of the PPMVs who participated in the study?*
2. Were the activities implemented in line with the plans discussed with the PPMVs association at the onset of the study? (*probe how well the project activities/processes were implemented?)*
3. Comment freely on quality and usefulness of the training on injectable service delivery
4. *Your competency on the delivery of injectable*
5. *Training and refresher, usefulness of the training you received on the delivery of injectable contraceptive*

Did the competency acquired through the training and other activities on the project translate into utilization of service

1. Comment freely on Injectable service delivery by PPMVs
2. *Experiences on the provision of injectable contraceptives services to clients with emphasis on willingness and attitude of women to receive injectable contraceptives from PPMVs*
3. *Availability and use of refresher instructional materials after training*
4. *Availability and use of IEC materials(job aids)*
5. *Injectable supply/re-supply and stock out*
6. *Consumables supply/re-supply and stock out*
7. *Availability and adherence of PPMVs to injectable service delivery guidelines*
8. *Monitoring, technical assistance*
9. Explain the extent to which women utilized services provided by the PPMVs. P*robe*
   1. Type of women who utilized the services by age and other socio-demographic characteristics
   2. *Referral to higher level facilities for family planning services*
   3. Satisfaction of injectable users?
   4. Extent to which service delivery by PPMVs was of enough quality
   5. *Referral to higher level facilities for family planning services*

Did the training influence satisfaction in general for e.g did more women patronize the PPMVs because they were trained. Did PPMVs do things differently post training that the women liked ? if yes, what were

1. What were the components of the intervention (*training, linkage to injectable commodity supply, technical assistance, monitoring, referral, enabling policy and regulatory environment etc*) which increased injectable service provision?
2. In your opinion, describe the most significant achievements on this project?
   1. Probe - what was the best feature of this intervention (*training, linkage to injectable commodity supply, technical assistance, monitoring, referral, enabling policy and regulatory environment and any etc)*
3. What were the major obstacles experienced during the implementation of the project if any?
4. Probe if injectable commodities were received on time?
5. Probe if technical assistance was received on time?
6. Probe for regulatory/policy support for the project (i.e.where they harassed by NDLEA, StateMinistry of Health)
7. What challenges did you encounter during project implementation that affected the provision of injectable services ?

**Mechanism Evaluation**

1. **What are the factors (***i.e. strengthened supervision, trained and knowledgeable PMVs***) which explain/influenced** the utilization of injectable contraceptive services**?**
   1. Which **factors** were most important to the success of the project (what works well)?
   2. Which **activities** were most critical to the failure of the project (what did not work well)?
   3. Which potential **activities/factors** if included in the study will likely influence the success or failure of the project ?
2. Were there any unintended impacts or consequences - either positive and/or negative?

Probe

1. Unintended impacts or consequences on the Injectable users/women (positive and/or negative)
2. Unintended impacts or consequences on the Community (positive and/or negative)
3. Unintended impacts or consequences on the PPMVs or their associations (positive and/or negative)
4. Unintended impacts or consequences on the Health system (positive and/or negative)

**Assessing Contextual Factors**

1. How did stakeholders **(***state officials of the Ministries of Health, Pharmacy Council of Nigeria, PPMVs, Police Force, women, and communities*) interact on the intervention?
   1. *Probe for the stakeholder’s views/opinions on appropriateness and acceptability of PPMVs providing injectable contraceptives*
   2. *Probe for the effects of their interactions or opinions on the project implementation and outcomes*
2. What were the factors external to the PPMVs intervention that may have influenced its implementation?
   1. Probe for community, health system and regulatory factors (pharmacy unit of the State Ministry of Health and the police force)
3. What were the factors external to the PPMVs intervention that may have influenced its outcomes?
   1. Probe for community, health system and regulatory factors (pharmacy unit of the State Ministry of Health and the police force)

**CLOSING**

Appreciate the respondents for their participation.

Remind them that the discussion will be kept confidential.

**Assessment of the Processes and Mechanisms of Influence of Proprietary Patent Medicine Vendors’ Training on Access to and Utilization of Injectable Contraceptives in Nigeria**

**Interview Guide for Health Workers in Government or Private Facilities**

**Note to Interviewer: *Share the study information sheet and request for signed consent***

**Demographics, TAKE NOTES** (identifying information to be kept separate from interview transcripts)

Interview #___________________

**Background Information**

1. Gender
2. Age at last birthday
3. Highest educational qualification
4. Religion
5. Participant’s organization and contact details (fill in beforehand if possible):
6. Participant’s title/designation and primary responsibilities:
7. What year did you start working in this organization? What year did you start in this position?

Questions

1. What type of family planning services does this facility provide? Probe for all methods –*condoms, pills, IUCDs, implants, vasectomy, bilateral tubal ligation etc.*
2. How would you describe the demand and utilization of family planning services among people in this community? *Probe*
3. *number of people, on average which use family planning services at the facility each week*
4. Probe for more details on profile of users e.g. married/unmarried, wealth status, men/women, old/young, ethnicity/religion
5. Comment freely on your role and involvement on the project. Probe for involvement:
6. in training of PPMVs on injectable contraceptive service delivery
7. Supply or-resupply of commodities
8. Two-way referral
9. Monitoring and provision of technical assistance
10. E. Disposal of sharpbox
11. Is there any linkage between the PPMV shops and your facility?

*[Comment on the linkage of PPMVs to the* formal private and public health care system*, the referral system used? Is there a back-referral system in place so the PPMV is aware of the services clients received at the health facility*, *if yes, how does that work?]*

1. If there is a referral linkage, has this influenced family planning service provision in your facility?

*Probe if this has increased, decreased or had no effect on family planning service provision since the inception of the project? Probe further on its effect on the provision of prescriptive family planning methods and other long acting permanent family planning methods*

1. Do you have any additional comments on successes, challenges or suggestions for improving the PPMV intervention?

**CLOSING**

Appreciate the respondents for their participation. Remind them that the discussion will be kept confidential.

**Assessment of the Processes and Mechanisms of Influence of Proprietary Patent Medicine Vendors’ Training on Access to and Utilization of Injectable Contraceptives in Nigeria**

**Key informant interview guide for Programme Implementers**

**Note to Interviewer: *Share the study information sheet and request for signed consent***

**Demographics, TAKE NOTES** (identifying information to be kept separate from interview transcripts)

Interview #___________________

**Background Information**

1. Gender
2. Age at last birthday
3. Highest educational qualification
4. Religion
5. Participant’s organization and contact details (fill in beforehand if possible):
6. Participant’s title/designation and primary responsibilities:
7. What year did you start working in this organization? What year did you start in this position?

**REQUEST TO TURN ON RECORDERS AT THIS POINT IN THE INTERVIEW**

1. What role did you play in the PPMV project?
2. How long did you work on the PPMV project?

**Implementation Evaluation**

1. In your own words, describe the PPMV project
2. Describe the main activities that you implemented, ***What happened? What was your role?*** (*probe for the training for PPMVs, commodity supply/re-supply and management, monitoring, evaluation activities etc.)*
3. Were the activities implemented as planned in the study proposal? (*probe how well the project activities/processes were implemented compared with quality standards?)*

If yes, what are the factors which enhance effective implementation?

If no, what are the reasons?

1. Were the alterations made to the PPMV intervention to adapt it to the Nigerian context, and to achieve the intended objectives? If yes, please explain in detail the alterations made, If no why?
2. Were outputs delivered on time? P*robe*
   1. *Number of PPMVs trained*
   2. *Competency of trained PPMVs*
   3. *Availability and adherence of PPMVs to injectable service delivery guidelines*
   4. *Availability and use of IEC materials*
   5. *Availability and use of instructional materials (job aids) to PPMVs*
   6. *Availability and use oif training manuals*
   7. *Injectable supply/re-supply and stock out*
   8. *Consumables supply/re-supply and stock out*
   9. *Monitoring, technical assistance*

*If no, probe for the reasons*

1. What were the components of the intervention which made a difference to the achievement of the study outcomes?
   1. Probe if the interaction between different activities contributed to achieving better outcomes.
2. Explain the extent to which women utilized services provided through the intervention. P*robe*
   1. Type of women who accessed the services who utilized the services by age and other socio-demographic characteristics
   2. Are women reached as intended? *(how many women were provided injectables? how many were referred to higher level facilities for family planning services)*
   3. How satisfied were the injectable users?
   4. Extent to which service delivery by PPMVs was of enough quality
3. In your opinion, describe the most significant achievements on this project?
   1. Probe what was the best feature of this intervention
4. What were the major obstacles experienced during the implementation of the project if any?
   1. Probe if fund was received on time?
   2. Probe if technical assistance was received on time?
   3. Probe for regulatory/policy support for the project
5. What emerging issues around implementation hindered progress towards the expected outcomes?

**Mechanism Evaluation**

1. **What are the factors that contributed to success of the project? (***probe for processes i.e. strengthened supervision, trained and knowledgeable PMVs*) which explain/influence the utilization of injectable contraceptive services?
   1. Which factors were most critical to the success of the project?
   2. Which activities/factors were most critical to the failure of the project?
   3. Which potential activities/factors if included in the study will likely influence the success or failure of the project?
2. Were there any unintended impacts or consequences - either positive and/or negative?

Probe

1. Unintended impacts or consequences on the Injectable users (positive and/or negative)
2. Unintended impacts or consequences on the Community (positive and/or negative)
3. Unintended impacts or consequences on the PPMVs or their associations (positive and/or negative)
4. Unintended impacts or consequences on the Health system (positive and/or negative)

**Assessing Contextual Factors**

1. How did the different stakeholders **(***federal and state officials of the Ministries of Health, Pharmacy Council of Nigeria,* Palladium-DMPA-SC scale-up project, *PPMVs Association, PPMVs, Health workers, Police Force, women, and communities*) interacted on the project?
   1. *Probe for the stakeholder’s views/opinions on appropriateness and acceptability of PPMVs providing injectable contraceptives*
   2. *Probe for the effects of their interactions or opinions on the project implementation and outcomes*
2. What were the factors external to the PPMVs intervention that may have influenced its implementation?
   1. Probe for community, health system, political and regulatory factors
   2. Donor and government performance in terms of making funds and other support available
3. What were the factors external to the PPMVs intervention that may have influenced its outcomes?
   1. Probe for community, health system, political and regulatory factors
   2. Donor and government performance in terms of making funds and other support available

**Assessment of the Processes and Mechanisms of Influence of Proprietary Patent Medicine Vendors’ Training on Access to and Utilization of Injectable Contraceptives in Nigeria**

**Key informant interview guide for officials of NAPPMED**

**Note to Interviewer: *Share the study information sheet and request for signed consent***

**Demographics, TAKE NOTES** (identifying information to be kept separate from interview transcripts)

Interview #___________________

**Background Information**

1. Gender
2. Age at last birthday
3. Highest educational qualification
4. Religion
5. Participant’s organization and contact details (fill in beforehand if possible)
6. Participant’s title/designation and primary responsibilities
7. What year did you start working in this organization? What year did you start in this position?

**REQUEST TO TURN ON RECORDERS AT THIS POINT IN THE INTERVIEW**

**Implementation Evaluation**

1. In your own words, describe the activities implemented on the project. *What happened? What role did you play in the project?*

*Probe for involvement in*

- 1. *role in selection of participants*
  2. *training for PPMVs*
  3. *commodity supply/re-supply and management*
  4. *monitoring and evaluation activities etc.*

1. Were the activities implemented in line with the plans agreed upon in discussions with the PPMV association (NAPPMED) at the onset of the study? (*probe how well the project activities/processes were implemented compared with quality standards and timelines?)*
   1. *If yes, what are the factors which enhanced effective implementation?*
   2. *If no, what are the reasons?*
2. Were outputs delivered on time? P*robe*
3. *Number of PPMVs trained*
4. *Competency of trained PPMVs*
5. *Availability and adherence of PPMVs to injectable service delivery guidelines*
6. *Availability and use of IEC materials*
7. *Availability and use of instructional materials to PPMVs*
8. *Injectable supply/re-supply and stock out*
9. *Consumables supply/re-supply and stock out*
10. *Monitoring, technical assistance*

*If no, probe for reasons*

1. What were the components of the intervention which made a difference to the achievement of the study outcomes?
2. Explain the extent to which women utilized services provided by the PPMVs. P*robe*
   1. Type of women who utilized the services by age and other socio-demographic characteristics
   2. Were the women reached as intended? *(how many women were provided injectables? how many were referred to higher level facilities for family planning services)*
   3. Satisfaction of users of the injectable contraceptives
   4. Supply of quantity of commodities to PPMV?
3. In your opinion, describe the most significant achievements on this project?
   1. Probe what was the best feature of this intervention
4. What were the major obstacles experienced during the implementation of the project if any?
5. Probe supply of injectables?
6. Probe if technical assistance was received on time?
7. Probe for regulatory/policy support for the project?
8. Provision of services to women?
9. What emerging issues around implementation hindered progress towards the expected outcomes?

**Mechanism Evaluation**

1. **What are the factors (***i.e. strengthened supervision, trained and knowledgeable PPMVs***) which explain/influenced** the utilization of injectable contraceptive services**?**
   1. Which **factors/activities** were most critical to the success of the project?
   2. Which **factors/ /activities** were most critical to the failure of the project?
   3. Which potential **factors/activities** if included in the study will likely influence the success or failure of the project?
2. Were there any unintended impacts or consequences - either positive and/or negative?

Probe

1. Unintended impacts or consequences on the Injectable users/women (positive and/or negative)
2. Unintended impacts or consequences on the Community (positive and/or negative)
3. Unintended impacts or consequences on the PPMVs or their associations (positive and/or negative)
4. Unintended impacts or consequences on the Health system (positive and/or negative)

**Assessing Contextual Factors**

1. How did stakeholders **(***state officials of the Ministries of Health, Pharmacy Council of Nigeria, PPMVs, Police Force, women, and communities*) interacted on the intervention?
   1. *Probe for the stakeholder’s views/opinions on appropriateness and acceptability of PPMVs providing injectable contraceptives*
   2. *Probe for the effects of their interactions or opinions on the project implementation and outcomes*
2. What were the factors external to the PPMVs intervention that may have influenced its implementation?
   1. Probe for community, health system and regulatory factors (pharmacy unit of the State Ministry of Health and the police force)
3. What were the factors external to the PPMVs intervention that may have influenced its outcomes?
   1. Probe for community, health system and regulatory factors (pharmacy unit of the State Ministry of Health and the police force)

**Closing: We thank you for answering our questions**

**Assessment of the Processes and Mechanisms of Influence of Proprietary Patent Medicine Vendors’ Training on Access to and Utilization of Injectable Contraceptives in Nigeria**

**Key informant interview guide for officials of government regulatory agencies**

**Note to Interviewer: *Share the study information sheet and request for signed consent***

**Demographics, TAKE NOTES** (identifying information to be kept separate from interview transcripts)

Interview #___________________

**Background Information**

1. Gender
2. Age at last birthday
3. Highest educational qualification
4. Religion
5. Participant’s organization and contact details (fill in beforehand if possible)
6. Participant’s title/designation and primary responsibilities
7. What year did you start working in this organization? What year did you start in this position?

**REQUEST TO TURN ON RECORDERS AT THIS POINT IN THE INTERVIEW**

**Implementation Evaluation**

1. In your own words, describe the activities implemented on this study, *what happened? What was your role?*

*Probe for involvement in*

- 1. *selection of study participants*
  2. *training for PPMVs*
  3. *commodity supply/re-supply and management*
  4. *monitoring and evaluation activities etc.*
  5. *Regulatory roles and functions during project implementation*

1. Were the activities implemented in line with the proposal as discussed with your agency prior the onset of the study? (*probe how well the project activities/processes were implemented compared with quality standards, timelines and regulations governing the PPMVs)*
2. If yes, what are the factors which enhanced effective implementation
3. If no, what are the reasons?
4. Were outputs delivered on time? P*robe*
   1. *Number of PPMVs trained*
   2. *Competency of trained PPMVs viz-a-viz national guidelines and standards for injectable contraceptive service delivery*
   3. *Availability and adherence of PPMVs to national guidelines and standards for injectable service delivery*
   4. *Availability and use of instructional materials to PPMVs*
   5. *Injectable supply/re-supply and stock out*
   6. *Consumables supply/re-supply and stock out*
   7. *Monitoring, technical assistance*

*If no, what are the reasons?*

1. What were the components of the intervention which made a difference to the achievement of the study outcomes?
2. What were the major obstacles experienced during the implementation of the project if any?
   1. Probe for funding, technical assistance, regulatory/policy support for the project or otherwise
3. What emerging issues around implementation hindered progress towards the expected outcomes?

**Mechanism Evaluation**

1. **What are the factors (***i.e. strengthened supervision, trained and knowledgeable PMVs***) which explain/influenced** the utilization of injectable contraceptive services provided by PPMVs**?**
   1. Which **factors** were most critical to the success of the project?
   2. Which **factors/activities** were most critical to the failure of the project?
   3. Which potential **activities** if included in the study will likely influence the success or failure of the project?
2. Were there any unintended impacts or consequences - either positive and/or negative?

Probe

1. Unintended impacts or consequences on the PPMVs and their associations (positive and/or negative)
2. Unintended impacts or consequences on the Health system (positive and/or negative)
3. Unintended impacts or consequences on your agency’s regulatory functions or roles (positive and/or negative)

**Assessing Contextual Factors**

1. How did the different stakeholders **(***federal and state officials of the Ministries of Health, Pharmacy Council of Nigeria, PPMVs Association, PPMVs, Health workers, Police Force, women, and communities*) interacted on the project?
   1. *Probe for the stakeholder’s views/opinions on appropriateness and acceptability*
   2. *Probe for the effects of their interactions or opinions on the project implementation and outcomes*
2. What were the factors external to the PPMVs intervention that may have influenced its implementation?
   1. Probe for community, health system, political and regulatory factors
   2. Donor and government performance in terms of making funds and other support available
3. What were the factors external to the PPMVs intervention that may have influenced its outcomes?
   1. Probe for health system, political and regulatory factors
   2. Donor and government performance in terms of making funds and other support available

**CLOSING**

Appreciate the respondents for their participation.

Remind them that the discussion will be kept confidential.
